# Secondary Metabolites from *Rehmannia glutinosa* Protect Mitochondrial Function in LPS-Injured Endothelial Cells

**DOI:** 10.3390/ph18081125

**Published:** 2025-07-27

**Authors:** Liwen Zhong, Mengkai Lu, Huiqi Fang, Chao Li, Hua Qu, Gang Ding

**Affiliations:** 1 State Key Laboratory of Bioactive Substances and Function of Natural Medicines, Institute of Medicinal Plant Development, Chinese Academy of Medical Sciences and Peking Union Medical College, Beijing 100193, China; s2023009049@student.pumc.edu.cn (L.Z.); klxsxjsw@163.com (H.F.); 2 College of Traditional Chinese Medicine, Shandong University of Traditional Chinese Medicine, Jinan 250355, China; mengkailu0912@163.com; 3 Xiyuan Hospital, China Academy of Chinese Medical Sciences, Beijing 100091, China; hua_qu@yeah.net

**Keywords:** *Rehmannia glutinosa* Libosch., eremophilane-type sesquiterpenes, UPLC-Q-TOF-MS, characteristic fragmentation pathways, LPS-induced HUVECs injury

## Abstract

**Background:** *Rehmannia glutinosa*, a traditional Chinese herb, is commonly used to treat vascular-related disorders. Sepsis-associated vascular endothelial dysfunction is closely associated with mitochondrial damage. This study investigated the protective effects of secondary metabolites from *R. glutinosa* against LPS-induced mitochondrial dysfunction in endothelial cells, providing potential therapeutic insights into sepsis-related vascular complications. **Methods**: Phytochemical profiling of fresh *R. glutinosa* roots was conducted, and the structures of new secondary metabolites (**1** and **2**) were elucidated through comprehensive spectroscopic analysis and ECD calculations. UPLC-Q-TOF-MS/MS characterized phenylethanoid glycosides. Mitochondrial function was assessed by measuring the membrane potential, ROS levels, and TOM20/DRP1 expression in LPS-injured HUVECs. **Results**: Two novel eremophilane-type sesquiterpenes, remophilanetriols J (**1**) and K (**2**), along with five known phenylethanoid glycosides (**3**–**7**), were isolated from the fresh roots of *R. glutinosa*. UPLC-Q-TOF-MS/MS analysis revealed unique fragmentation pathways for phenylethanoid glycosides (**3**–**7**). In LPS-injured HUVECs, all compounds collectively restored the mitochondrial membrane potential, attenuated ROS accumulation, and modulated TOM20/DRP1 expression. In particular, remophilanetriol K (**2**) exhibited potent protective effects at a low concentration (1.5625 μM). **Conclusions**: This study identifies *R. glutinosa* metabolites as potential therapeutics for sepsis-associated vascular dysfunction by preserving mitochondrial homeostasis. This study provides a mechanistic basis for the traditional use of *R. glutinosa* and offers valuable insights into the development of novel therapeutics targeting mitochondrial dysfunction in sepsis.

## 1. Introduction

*Rehmannia glutinosa* Libosch., a classical medicinal herb belonging to the Scrophulariaceae family, was first recorded in *Shennong’s Classic of Materia Medica* for its efficacy in “dispelling blood stasis and replenishing marrow essence” [1]. Contemporary pharmacological investigations have demonstrated that fresh *R. glutinosa* rhizomes contain various bioactive constituents that possess significant antioxidant, anti-inflammatory, and vasculoprotective properties [2,3]. These pharmacological properties are consistent with its traditional indications for vascular-related disorders, such as heat entering the nutritive blood level, and hemorrhagic symptoms, including macules, eruptions, hematemesis, and epistaxis [4].

In recent years, studies have demonstrated that sepsis-associated vascular endothelial dysfunction serves as a pivotal contributor to pathological conditions, such as circulatory instability, immune dysregulation, and multi-organ dysfunction [5,6]. Lipopolysaccharide (LPS)-triggered mitochondrial impairment intensifies endothelial barrier damage by promoting excessive reactive oxygen species (ROS) generation and disrupting energy metabolism [7,8,9]. While previous studies have demonstrated the efficacy of *R. glutinosa* extracts in mitigating sepsis-associated renal injury [10] and the clinical utility of related compound formulations such as Xijiao Dihuang Decoction [11], the specific effects of its bioactive constituents on LPS-induced vascular endothelial injury remain unknown.

To bridge this knowledge gap, phytochemical profiling of fresh *R. glutinosa* roots was conducted, leading to the isolation and structural characterization of two novel eremophilane-type sesquiterpenes (**1**–**2**) and five known phenylethanol glycosides (**3**–**7**). Eremophilane-type sesquiterpenes, characterized by a *trans*-decalin core bearing a transannular isopropenyl group, are predominantly reported in the Asteraceae family, with only a limited number identified in the Scrophulariaceae family [12]. To date, merely 12 structurally distinct eremophilane-type sesquiterpenes have been isolated from *R. glutinosa* (Table S1). Existing evidence indicates that eremophilane-type sesquiterpenes and phenylethanol glycosides exhibit diverse pharmacological properties, including antibacterial, anti-inflammatory, and antioxidant activities, implying their potential therapeutic value in the management of sepsis [13,14]. To further characterize the pharmacological potential of these compounds, an LPS-induced injury model in human umbilical vein endothelial cells (HUVECs) was employed to systematically evaluate the effects of these compounds on mitochondrial morphology, membrane potential dynamics, reactive oxygen species (ROS) accumulation, and expression patterns of key mitochondrial proteins. These results provide new insights into the classical “cooling blood and dispersing stasis” action of *R. glutinosa* and reveal potential molecular targets that could inform the future development of mitochondria-targeted therapies for sepsis-associated vascular dysfunction.

## 2. Results and Discussion

### 2.1. Structure Determination

Compound **1**, with a chemical composition of C_15_H_22_O_2_, was established based on HR-ESI-MS ([M + H]^+^, *m/z* 235.1708, calcd. for 235.1698), indicating five degrees of unsaturation in the structure (Figure S7). The IR spectrum of **1** exhibited characteristic absorptions for conjugated carbonyl (1666 cm^−1^), methylene (2925 and 2854 cm^−1^), and hydroxyl (3363 cm^−1^) groups (Figure S8). A comprehensive analysis of the ^1^H NMR and HSQC spectra (Figures S1 and S4) revealed that the ^13^C NMR spectrum of **1** (Table 1) exhibited a total of fifteen carbon signals, including two methyls (*δ*_C_ 11.9, 15.4), six methylenes (alkyl carbons at *δ*_C_ 20.7, 25.3, 30.2, 41.1; O-substituted carbon at *δ*_C_ 63.8 and olefinic carbon at *δ*_C_ 117.3), three methines (alkyl carbons at *δ*_C_ 43.3, 55.2; olefinic carbon at *δ*_C_ 124.0), and four quaternary carbons (alkyl carbon at *δ*_C_ 40.0; olefinic carbons at *δ*_C_ 146.4, 151.8 and carbonyl carbon at *δ*_C_ 201.9). In addition to featuring one carbonyl and two double bonds, the bicyclic framework of **1** was essential for fulfilling the unsaturation degree requirement.

**Figure 1:**
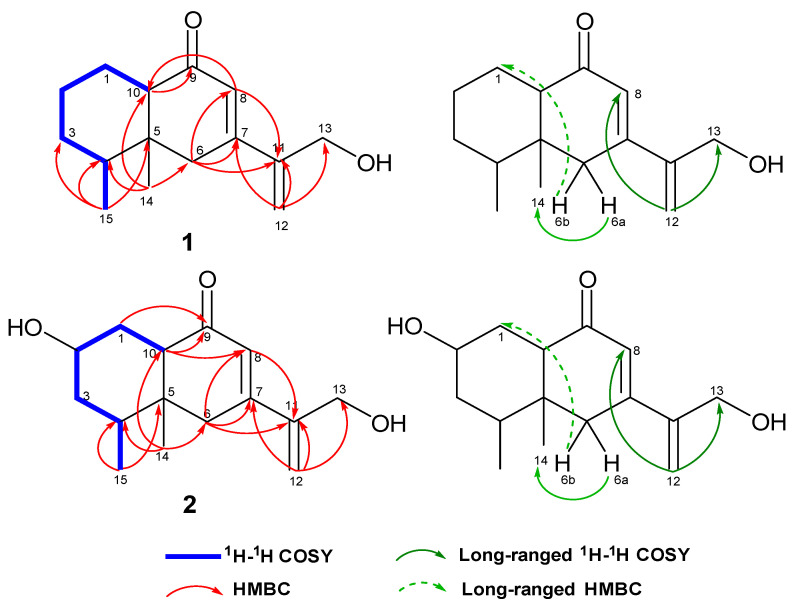
Key 2D-NMR correlations for compounds **1** and **2**.

Based on the ^1^H–^1^H COSY correlations (Figure 1 and Figure S3) of H-10/H_2_-1/H_2_-2/H_2_-3/H-4/H_3_-15, the connection of C-10/C-1/C-2/C-3/C-4/C-15 was confirmed. The HMBC correlations from H_3_-15 to C-5 and from H_3_-14 to C-4/C-5/C-10 corroborated the connection of C-5 to C-4/C-10/C-14, constructing the ortho-dimethyl-substituted cyclohexane unit of compound **1**. The attachment between C-6 and C-5 was deduced from the HMBC correlations between H_3_-14 and C-6. The HMBC correlations between H_2_-12 and C-11/C-13/C-7 confirmed the presence of an oxymethyl-substituted terminal alkenyl. The attachment between C-7 and C-6/C-8/C-11 was verified through the HMBC correlations from H_b_-6 (*δ*_H_ 2.68) to C-7/C-11/C-8 and from H-8 to C-11. Notably, the ^1^H−^1^H COSY data indicated significant long-range correlations from H_2_-12 (*δ*_H_ 5.59, 5.60) to both H_2_-13 (*δ*_H_ 4.38) and H-8 (*δ*_H_ 6.05), and the signal of H_2_-12/H_2_-13 was stronger than H_2_-12/H-8, providing additional valuable information for elucidating the structure (Figure 1). The attachments between C-9 and C-10/C-8 to form another closed ring were inferred from the HMBC correlations between H-10 and C-9 and between H-8 and C-10. Moreover, the C-8/H-8 in **1** exhibited a significant downfield shift attributed to the deshielding effect caused by the carbonyl group. A hydroxyl group was proposed to be attached to C-13 (δC 63.8) based on the molecular formula C_15_H_22_O_2_. The planar structure of compound **1**, analogous to remophilanetriol [15], an eremophilane-type metabolite isolated from *R. glutinosa*, was established (Figure 1).

**Figure 2:**
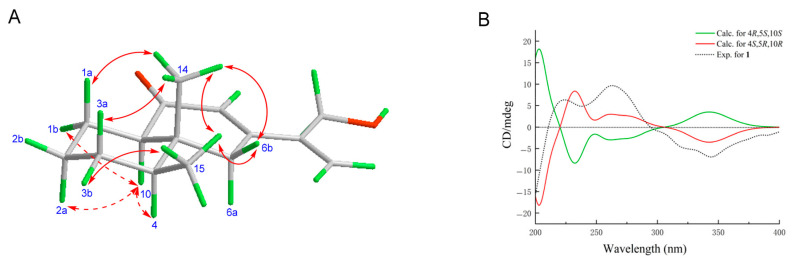
Key NOE correlations of **1** (**A**) and calculated and experimental ECD spectra of **1** (**B**).

**Figure 3:**
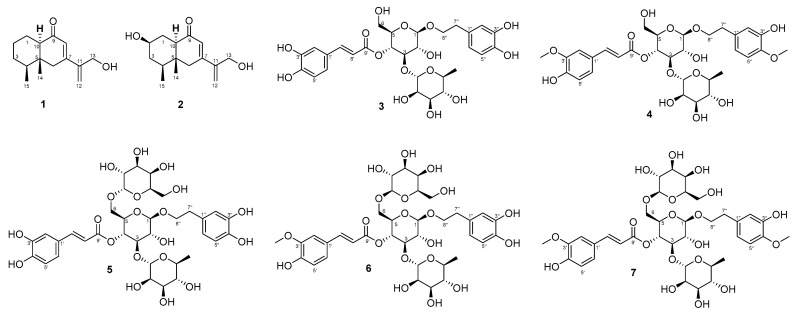
Structures of **1**−**7**.

The relative configuration of **1** was established by analyzing the coupling constants and conducting NOE experiments. The observed NOE effects from CH_3_-14 (*δ*_H_ 0.77) to CH_3_-15 (*δ*_H_ 0.90)/H_a_-1 (*δ*_H_ 1.29)/H_a_-3 (*δ*_H_ 1.25)/H_b_-6 (*δ*_H_ 2.68), as well as from H-10 (*δ*_H_ 2.21) to H_a_-6 (*δ*_H_ 2.30)/H_b_-1 (*δ*_H_ 2.00)/H-4 (*δ*_H_ 1.55)/H_a_-2 (*δ*_H_ 1.30), indicated that CH_3_-14/CH_3_-15/H_a_-1/H_b_-2/H_a_-3/H_b_-6 were situated on one side of the structure, while H-10/H_b_-1/H_a_-2/H_b_-3/H-4/H_a_-6 were positioned on the opposite side (Figure S6 and Figure 2A). The *trans* configuration between H-10 and CH_3_-14 revealed a *trans*-decalin structure for **1**. Calculations using Chem3D demonstrated that the chair conformation, with CH_3_-14 and H-10 oriented axially, was essential to satisfy the energy minimization criteria and the coupling constant of 11.5 Hz for H-10 (Figure 2A and Table 1) [15]. Accordingly, the groups H_a_-1/H_a_-2/H_a_-3/H-4/H_a_-6 were positioned axially. Furthermore, the observed *w*-type long-range correlation between H_a_-6 (*δ*_H_ 2.30) and H_3_-14 (*δ*_H_ 0.77) in the ^1^H−^1^H COSY spectrum, as well as the *w*-type long-range coupling from H_b_-6 (*δ*_H_ 2.68) to C-1 (*δ*_c_ 20.7) in the HMBC spectrum, further substantiates this conclusion (Figure 1). The absolute configuration of **1** (4*S*, 5*R*, 10*R*) was established by comparing the experimental and TD-DFT-calculated (B3LYP/6-311+G (2d,p)) ECD spectra (Figure 2B and Figure 3) [16,17].

Compound **2** (C_15_H_22_O_3_, five degrees of unsaturation) was identified by HR-ESI-MS ([M + H]^+^, calcd. 251.1647) (Figure S15). The ^13^C NMR and HMQC data of compound **2** revealed two methyls (*δ*_C_ 11.7, 14.9), five methylenes (*δ*_C_ 30.1, 39.1, 40.7, 63.4, 117.5), four methines (*δ*_C_ 41.4, 53.1, 69.9, 123.6), and four quaternary carbons (*δ*_C_ 38.9, 145.9, 151.9, 200.2), demonstrating a close structural similarity to compound **1** (Figures S11 and S13). Based on the ^1^H spectrum and 2D NMR data of compounds **2** and **1**, the primary structural difference is the presence of the OH group at C-2 (*δ*_H_ 3.70/*δ*_C_ 69.9) (Table 1). The axial orientation of H-2 (with OH-2 in the equatorial position) was confirmed by the large axial-axial coupling constants (*J* = 11.5 Hz) observed in the ^1^H NMR spectrum, indicating that the OH-2 orientation was aligned with CH_3_-14 and CH_3_-15 (Table 1 and Figure 3). This evidence suggests the 2*R*, 4*S*, 5*R*, 10*R* absolute configuration of **2** (Figure 3). A comparison of the NMR data and shared biosynthetic origins between **2** and remophilanetriol B, a known compound from fresh *R. glutinosa* roots, further supports this conclusion [18].

In addition to compounds **1** and **2**, five known phenylethanoid glycosides were isolated, including acteoside (**3**) [19], martynoside (**4**) [20], purpureaside C (**5**) [20], jionoside A_1_ (**6**) [21], and jionoside B_1_ (**7**) [21]. The structural identities of these compounds were established by correlating their MS and NMR spectral characteristics with those reported in the existing literature.

### 2.2. Fragmentation Mechanisms of Compounds (***3***–***7***)

UPLC-Q-TOF-MS/MS analysis of acteoside (**3**) detected its protonated molecular ion ([M + H]^+^) at *m/z* 625, with a relatively low signal intensity. The fragment ions and elemental constituents combined with the high-resolution mass analysis of acteoside (**3**) are shown in Figure S18 and Table S2, respectively. According to mass analysis, the cleavage routes of **3** were described in Figure S23, in which typical charge-induced heterolysis, quadric rearrangement, and neutral loss were the main cleavage ways of compound **3** [22,23]. As shown in Figure S23, the ion (C_9_H_7_O_3_^+^, *m/z* = 163) of caffeoyl exhibited the highest relative abundance. The mass spectra indicated that this ion was primarily formed via two cleavage pathways. One was the parent ion *m/z* 625 of **3** by losing a rhamnose molecule (C_6_H_12_O_5_, −164 Da, quadric rearrangement) and a 3,4-dihydroxyphenylethanol molecule (C_8_H_10_O_3_, −154 Da, charge-induced heterolysis) in a specific sequence to yield the daughter ion (C_15_H_15_O_7_^+^, *m/z* = 307). Then the precursor ion *m/z* 307 produced the ion (*m/z* 127, C_6_H_7_O_3_^+^) and a caffeic acid molecule (180 Da) through rearrangement. The caffeic acid molecule (180 Da) was first ionized to produce protonated ions (*m/z* 181, C_9_H_9_O_4_^+^), and then lost one molecule of H_2_O (−18 Da) to generate the caffeoyl ion *m/z* 163. The other cleavage pathway indicated that the daughter ion of *m/z* 163 was formed from the parent ion *m/z* 625 by losing one rhamnosyl moiety (C_6_H_10_O_4_, -146 Da), one molecule of 3,4-dihydroxyphenylethanol (C_8_H_10_O_3_, −154 Da), and one inner glucosyl moiety (C_6_H_10_O_6_, −162 Da) in a specific order through charge-induced heterolysis. The relative abundance of the *m/z* 479 ion (9.58%) was higher than that of the *m/z* 471 ion (7.61%), indicating that the rhamnosyl group at C-3 was more easily lost than the phenethyl group at C-1. The base peak ion *m/z* 163 yields the fragment ion *m/z* 117 by losing CO (−28 Da) and H_2_O (−18 Da) in a specific sequence. It is worth mentioning that the caffeoyl ion (*m/z* 163, C_9_H_7_O_3_^+^) could not be directly yielded from the parent ion ([M + H]+, *m/z* 625) by losing the C_20_H_30_O_12_ molecule (−462 Da) through heterolysis, as the absence of *m/z* 463, 317 and 309 in the MS spectra indicates that caffeoyl at C-4 is more difficult to lose than the phenylethanol molecule at C-1 or rhamnosyl at C-3. The fragmentation pattern of martynoside (**4**) resembled that of acteoside (**3**), with methyl substitutions at the 3′-OH and 4″-OH positions, introducing a mass difference of 28 Da or 14 Da in the corresponding fragment ions (Table S3 and Figures S19 and S24).

**Figure 4:**
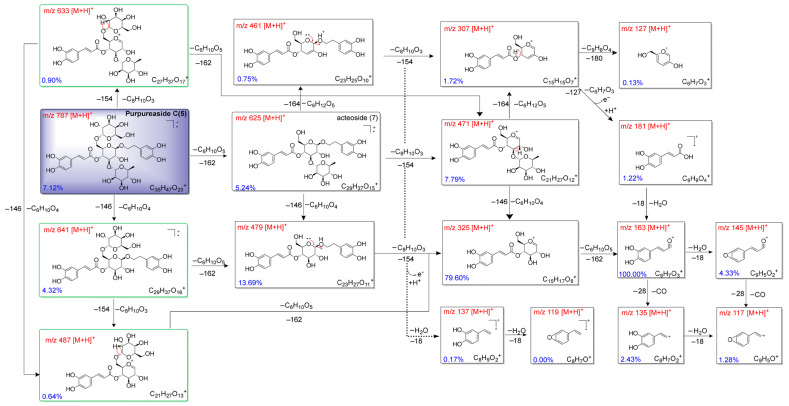
Possible mass fragmentation pathways of purpureaside C (**5**).

The ESI-MS spectra of purpureaside C (**5**) and acteoside (**3**) showed notable similarities in their fragmentation patterns (Figures S18 and S20). Specifically, the purpureaside C (**5**) ion *m/z* 787 (C_35_H_47_O_20_^+^) generated the acteoside (**3**) ion (*m/z* 625, C_29_H_37_O_15_^+^) by the loss of a galactosyl group (−162 Da), indicating an additional galactose in the structure of purpureaside C (**5**) (Figure 4). Compared to the cleavage pathway of acteoside (**3**), purpureaside C (**5**) exhibited an additional cleavage route for intermediate ions at *m/z* 471, 479, and 325. As depicted in Figure 4, the parent ion *m/z* 787 consequently lost C_8_H_10_O_3_ (−154 Da) and C_6_H_10_O_5_ (−162 Da) to yield *m/z* 471, and C_6_H_10_O_4_ (−146 Da) and C_6_H_10_O_5_ (−162 Da) in a specific sequence to yield *m/z* 479. The daughter ion of *m/z* 325 was derived from the parent ion *m/z* 787 by the loss of one rhamnosyl moiety (C_6_H_10_O_4_, −146 Da), one molecule of 3,4-dihydroxyphenylethanol (C_8_H_10_O_3_, −154 Da), and one galactosyl moiety (C_6_H_10_O_5_, −162 Da) in a certain order, through charge-induced heterolysis and quadric rearrangement. The ease of side chain moiety loss can be summarized as follows: loss of the galactosyl group at C-6 (−162 Da) > loss of the rhamnosyl at C-3 group (−146 Da) > loss of the phenethyl molecule at C-1 (−154 Da) > loss of the caffeoyl group at C-4 (−162 Da). This conclusion was based on the relative abundances of daughter ions *m/z* 625 (5.24%), 641 (4.32%), 633 (0.90%), and 471 (0.00%), which were derived from the parent ion (*m/z* 787, C_35_H_47_O_20_^+^) (Figure 4 and Table S4). Moreover, the presence of a galactosyl group at C-6 enhanced the relative intensities of the *m/z* 625, 479, and 325 ions in purpureaside C (**5**) compared to those in acteoside (**3**). The relevant data about elemental compositions and fragment ions of compound **5** are displayed in Table S4.

The abundance of the parent ion of jionoside A_1_ (**6**) ([M + H]^+^, *m/z* 801) was relatively low according to the mass profile (Figure S25). The relevant data regarding elemental compositions and fragment ions of compound **6** are presented in Table S5. The presence of the feruloyl group caused most of the fragment ions in the mass spectrum of jionoside A_1_ (**6**) to be 14 Da heavier than those in purpureaside C (**5**), except for *m/z* 127, 137, 119, 145, and 117. Notably, the *m/z* 145 (C_9_H_5_O_2_^+^) and *m/z* 117 (C_8_H_5_O^+^) ions in the ESI-MS spectrum of jionoside A_1_ (**6**) originated directly from the precursor ions *m/z* 177 and 149 by losing a methanol molecule (32 Da), respectively, bypassing intermediate ions such as C_9_H_7_O_3_^+^ (−14 Da, losing CH_2_) and C_10_H_7_O_3_^+^ (−2 Da, losing H_2_), because the *m/z* 163 and *m/z* 175 ions were absent from the spectrum (Figures S21 and S25 and Table S5). The cleavage pathways of jionoside B_1_ (**7**) were analogous to those of **6** (Table S6 and Figures S22 and S26).

### 2.3. Determination of Cytotoxic Effects and Experimental Concentrations of Compounds (***1***–***7***) in HUVECs

**Figure 5:**
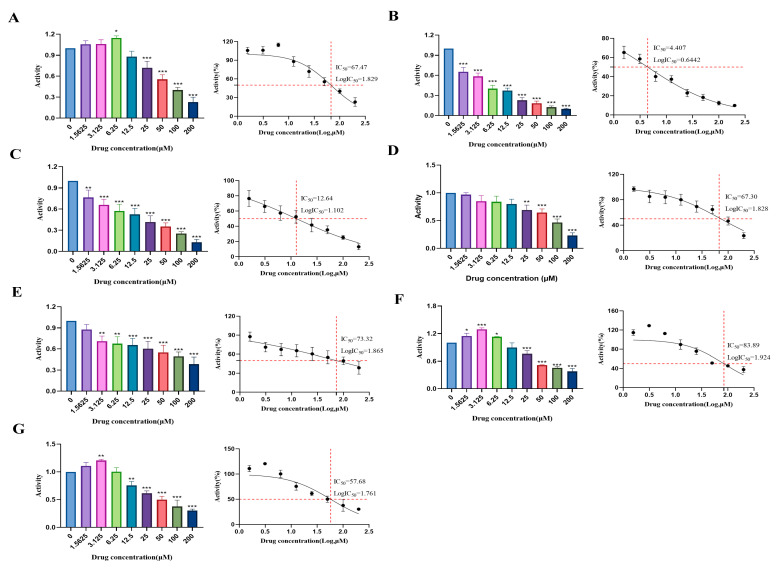
Cytotoxic effects (**A**–**G**) of compounds (**1**–**7**) on HUVECs (*n* = 3). The tests were performed in triplicate, and the data are presented as the mean ± SEM. Statistical significance is indicated as * *p* < 0.05, ** *p* < 0.01, and *** *p* < 0.001 vs. the drug-untreated control group.

The cytotoxicity of compounds (**1**–**7**) in HUVECs was evaluated using the CCK-8 assay to determine the experimental concentrations (Figure 5). Concentration-dependent viability effects were observed, with compounds **2**–**5** exhibiting progressive inhibition. Remophilanetriol J (**1**) and jionoside A_1_ (**6**) exhibited minimal effects at low concentrations but significant inhibition at higher doses (Figure 5).

Structural activity analysis revealed marked differences between the eremophilane-type metabolites. Remophilanetriol J (**1**) (IC_50_ = 67.47 μM) enhanced viability (1.56–6.25 μM), whereas the C-2 hydroxylated analog remophilanetriol K (**2**) showed significantly greater cytotoxicity (IC_50_ = 4.407 μM) (Figure 5 and Table S7). Among the phenylethanoid glycosides (**3**–**7**), methyl/glycosyl substitutions in compounds **4**–**7** may be correlated with reduced cytotoxicity compared to compound **3**.

Based on the dose–response profiles and IC_50_ values, three intervention concentrations (low, medium, and high) were selected for each compound (Table S8).

### 2.4. Effects of Diverse Compound Interventions on LPS-Induced Endothelial Cell Migration Impairment

**Figure 6:**
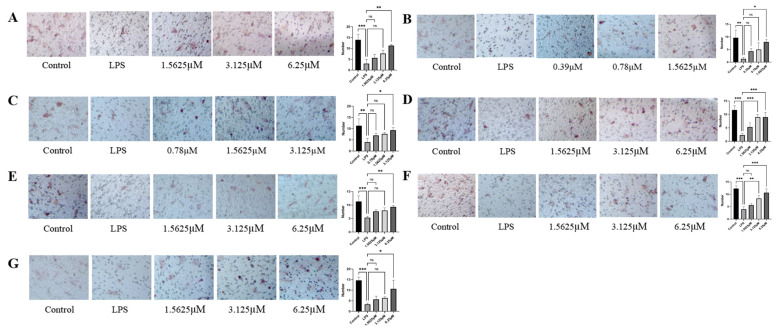
HUVEC staining results and quantitative analysis (**A**–**G**) of each compound (**1**–**7**) intervention on LPS-induced HUVEC migration capacity (*n* = 3). All images were captured at a magnification of 10×. Data are presented as mean ± SEM. Statistical significance is defined as * *p* < 0.05, ** *p* < 0.01, and *** *p* < 0.001, compared to the LPS-treated group.

LPS treatment significantly inhibited endothelial cell migration compared to the control (*p* < 0.01), establishing a valid model of migratory impairment in endothelial cells (Figure 6). As shown in Figure 6, all tested compounds enhanced the migration of LPS-injured HUVECs in a dose-dependent manner. The protective effects of compounds **1**–**7** against LPS-impaired HUVEC migration were quantified (Table S9). As shown in Table S9, at a concentration of 1.5625 μM, remophilanetriol K (**2**) (80.00 ± 6.93%) exhibited the most potent cytoprotective activity among the seven tested compounds, with acteoside (**3**) (50.00 ± 4.55%) showing the second highest efficacy.

### 2.5. Inhibitory Potency of Compounds Against ROS Levels in LPS-Induced HUVECs

**Figure 7:**
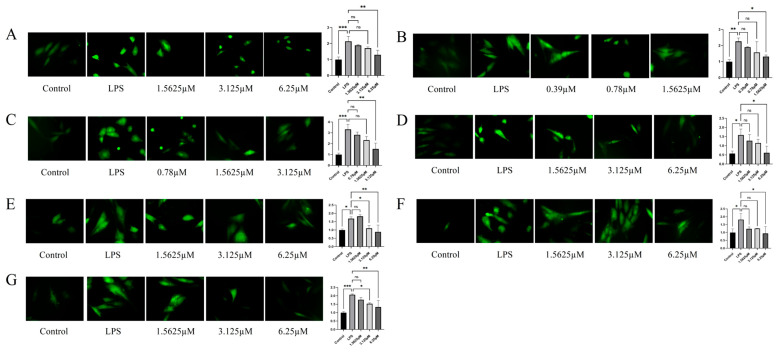
ROS staining results and quantitative analysis (**A**–**G**) of each compound (**1**–**7**) intervention in LPS-induced HUVECs (*n* = 3). All images were captured at a magnification of 20×. Data are presented as mean ± SEM. Statistical significance is indicated as * *p* < 0.05, ** *p* < 0.01, and *** *p* < 0.001 vs. the LPS-treated group.

The intracellular ROS levels in LPS-stimulated HUVECs were assessed for seven compounds (**1**–**7**). LPS treatment significantly increased ROS generation compared to that in the control group (*p* < 0.05). Dose-dependent ROS reduction was observed for most compounds, with purpureaside C (**5**) and jionoside B_1_ (**7**) demonstrating significant suppression at medium and high concentrations (*p* < 0.05, vs. LPS-treated group). All compounds exhibited significant inhibitory effects at high doses (*p* < 0.05, vs. LPS-treated group) (Figure 7 and Table S10). Comparative analysis at 1.5625 μM (Table S10) revealed that among the seven tested compounds, remophilanetriol K (**2**) (74.78 ± 4.56%) displayed the most potent ROS scavenging activity, followed by jionoside A_1_ (**6**) (71.69 ± 8.75%), and acteoside (**3**) (42.54 ± 8.39%) ranked third.

Structural activity analysis revealed significant differences in ROS modulation among the compounds despite their shared skeletons (Table S10). Remophilanetriol K (**2**) (1.56 μM, 74.8 ± 4.6%) demonstrated superior ROS clearance versus remophilanetriol J (**1**) (6.25 μM, 73.5 ± 14.1%), suggesting C-2 hydroxylation enhances antioxidant capacity. Among the phenylethanoid glycosides (**3**–**7**), martynoside (**4**) (6.25 μM, 83.6 ± 9.1%) showed significantly greater activity than jionoside B_1_ (**7**) (6.25 μM, 65.8 ± 18.4%), indicating that C-6 glycosylation reduces efficacy, potentially through impaired membrane permeability or target binding [24,25]. Comparative analysis at 3.125 μM confirmed structure-dependent activity: purpureaside C (**5**) (80.45 ± 10.01%) > jionoside A_1_ (**6**) (68.55 ± 0.40%) > jionoside B_1_ (**7**) (50.25 ± 3.05%) and acteoside (**3**) (76.43 ± 12.32%) > martynoside (**4**) (42.84 ± 11.52%), demonstrating that phenolic hydroxyl methylation decreases antioxidant capacity by disrupting molecular planarity and hydrogen bonding [25,26,27].

### 2.6. Effects of Compound Intervention on Mitochondrial Function in LPS-Induced HUVECs

**Figure 8:**
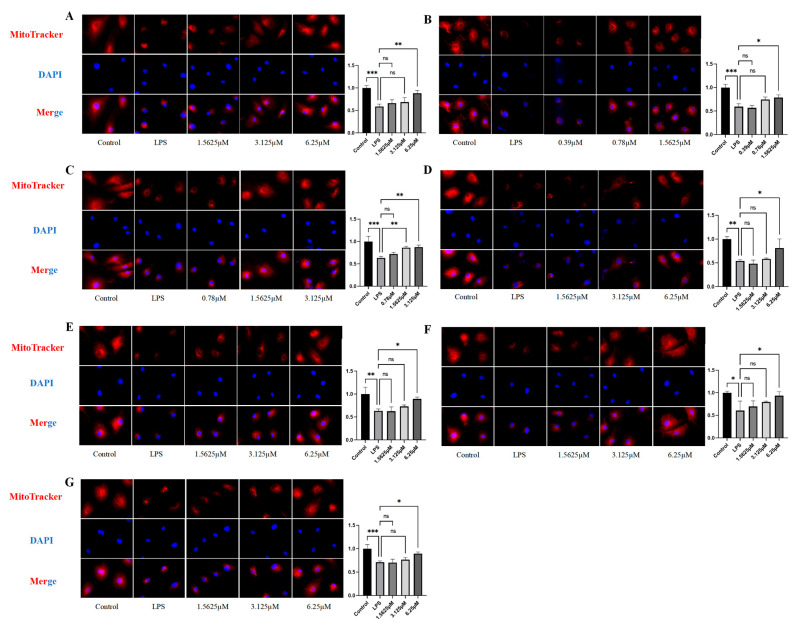
The Mitotracker staining results and fluorescence intensity quantitative analysis (**A**–**G**) of each compound (**1**–**7**) intervention on LPS-induced HUVECs (*n* = 3). All images were captured at a magnification of 20×. Data are expressed as mean ± SEM. Statistical significance is indicated as * *p* < 0.05, ** *p* < 0.01, and *** *p* < 0.001 vs. the LPS-treated group.

This study evaluated seven compounds (**1**–**7**) for their effects on mitochondrial activity in LPS-stimulated HUVECs using MitoTracker staining. LPS significantly reduced mitochondrial fluorescence intensity (*p* < 0.05) and induced a clustering-to-granulation transition (Figure 8), indicating damage [8]. Compared to the LPS-treated group, all compounds exhibited dose-dependent protection (p < 0.05) (Figure 8). Acteoside (**3**) showed significant protection at medium (1.56 μM, 62.17 ± 5.01%) and high doses (3.13 μM, 65.97 ± 7.04%) (*p* < 0.01). Comparative evaluation at 1.5625 μM revealed that acteoside (**3**) (62.17 ± 5.01%) exhibited the most significant cytoprotective effect among the seven compounds tested, with remophilanetriol K (**2**) (47.48 ± 8.24%) showing the second highest activity (Table S11).

**Figure 9:**
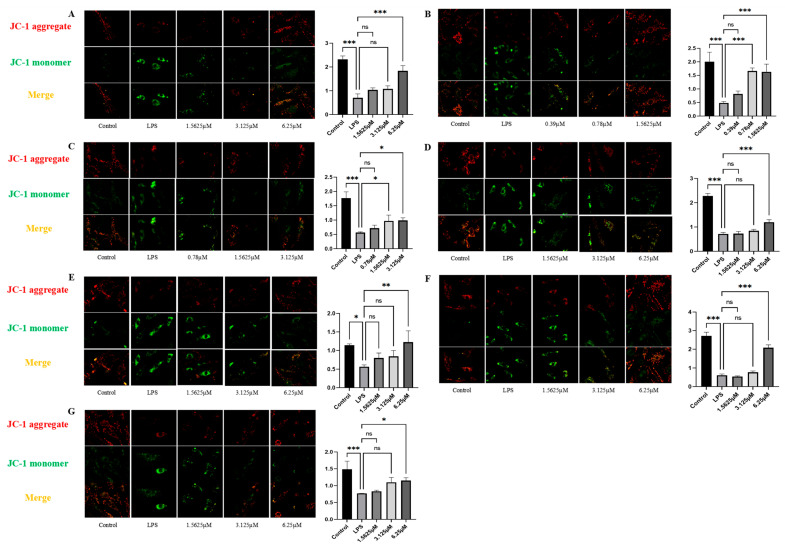
JC-1 staining results and bar charts of red/green fluorescence intensity ratios in LPS-induced HUVECs treated with each compound (**1**–**7**) (**A**–**G**), *n* = 3. All images were captured at a magnification of 20×. Data are displayed as mean ± SEM. Statistical significance is indicated as * *p* < 0.05, ** *p* < 0.01, and *** *p* < 0.001 vs. the LPS-treated group.

The mitochondrial membrane potential (Δ*Ψ*_m_), which is critical for cellular homeostasis [28], was assessed by JC-1 staining. JC-1 staining showed that LPS significantly decreased Δ*Ψ*_m_ (*p* < 0.05 vs. control), with a reduced red/green fluorescence ratio (Figure 9). All compounds significantly protected against mitochondrial depolarization at high doses (*p* < 0.05) (Table S12), with remophilanetriol J (**1**), remophilanetriol K (**2**), purpureaside C (**5**), jionoside A_1_ (**6**), and jionoside B_1_ (**7**) restoring Δ*Ψ*_m_ by more than 50%. Comparative evaluation at 1.5625 μM indicated that among the seven compounds tested, remophilanetriol K (**2**) exhibited the highest cytoprotective efficacy (75.36 ± 10.82%). Purpureaside C (**5**) and acteoside (**3**) ranked second and third, with activities of 40.63 ± 13.42% and 33.64 ± 9.77%, respectively (Table S12).

### 2.7. Effect of Compound Intervention on LPS-Induced Mitochondrial Homeostasis in Endothelial Cells

Thanslocase TOM20, which is essential for mitochondrial protein import and homeostasis [29,30,31], and DRP1, a key mediator of mitochondrial fission and dysfunction [32,33], were evaluated to assess the effects of the compounds on LPS-induced mitochondrial impairment.

**Figure 10:**
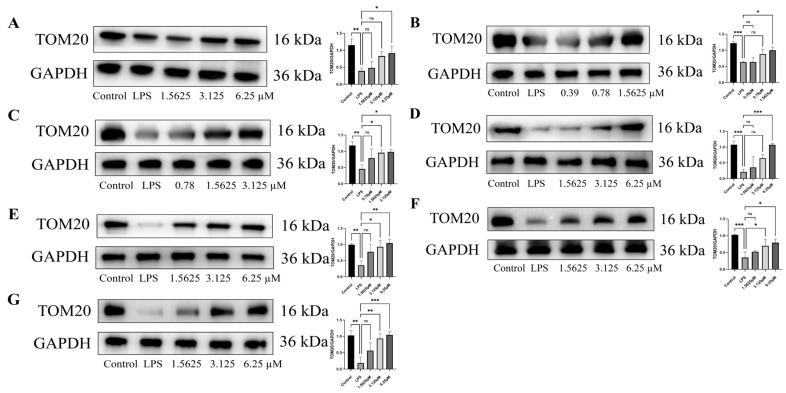
Effect of compounds (**1**–**7**) on LPS-induced TOM20 protein expression in endothelial cells (**A**–**G**), *n* = 3. Data are exhibited as mean ± SEM. Statistical significance is indicated as * *p* < 0.05, ** *p* < 0.01, and *** *p* < 0.001 vs. the LPS-treated group.

**Figure 11:**
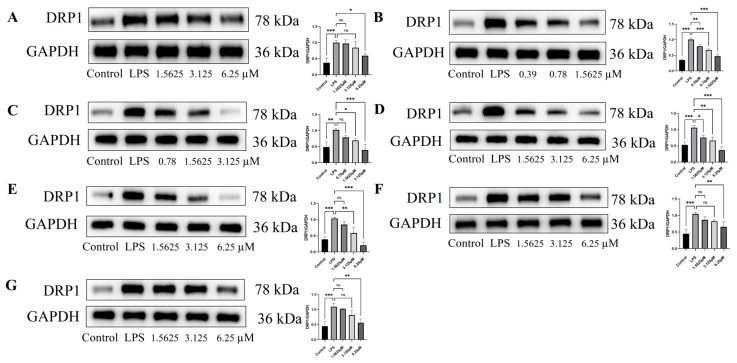
Effect of compounds (**1**–**7**) on LPS-induced DRP1 protein expression in endothelial cells (**A**–**G**), *n* = 3. Data are shown as mean ± SEM. Statistical significance is indicated as * *p* < 0.05, ** *p* < 0.01, and *** *p* < 0.001 vs. the LPS-treated group.

LPS treatment significantly impairs mitochondrial homeostasis in endothelial cells, as evidenced by decreased TOM20 expression and increased DRP1 levels compared to the control (*p* < 0.05) [32,33,34,35]. In HUVECs, all tested compounds significantly increased TOM20 expression and reduced DRP1 levels in a dose-dependent manner compared to the LPS-treated model group (*p* < 0.05), demonstrating potent protective effects on mitochondrial homeostasis (Figure 10 and Figure 11). Among all tested compounds (at 1.5625 μM, vs. LPS-treated model group), remophilanetriol K (**2**) and acteoside (**3**) exhibited superior mitochondrial protective effects, as evidenced by their ability to upregulate TOM20 expression (reversing LPS-induced suppression by 62.25 ± 9.69% and 69.18 ± 7.31%, respectively) and downregulate DRP1 levels (attenuating LPS-induced overexpression by 81.81 ± 5.68% and 61.25 ± 5.60%, respectively) (Tables S13 and S14).

Comparative analysis revealed that LPS-treated endothelial cells (model group) exhibited significantly impaired migratory capacity, elevated ROS levels, Δ*Ψ*_m_ depolarization, downregulated expression of the mitochondrial import protein TOM20, and upregulated expression of the fission-regulating protein DRP1 compared to the normal control group (*p* < 0.05). Existing evidence indicates that excessive ROS accumulation compromises mitochondrial integrity through multiple pathways, including membrane structural damage (manifested as Δ*Ψ*_m_ dissipation) and DRP1 overexpression, ultimately leading to mitochondrial dysfunction and subsequent cellular apoptosis [33,36]. The experimental observations of concurrent ROS elevation, Δ*Ψ*_m_ depolarization, and DRP1 upregulation in LPS-stimulated endothelial cells demonstrate a mechanistic correlation among these pathological alterations.

Mitotracker staining analysis (Figure 8) demonstrated that LPS-treated endothelial cells exhibited fragmented mitochondrial morphology, likely resulting from excessive mitochondrial fission mediated by upregulated DRP1 expression [32,37]. Mitochondrial fragmentation represents a hallmark of disrupted mitochondrial homeostasis, which in turn promotes excessive ROS generation and impairs both mitochondrial energy production and normal endothelial cell function [32,37]. Concurrently, the observed downregulation of TOM20 protein expression compromises mitochondrial protein import machinery, leading to disturbances in energy metabolism and quality control mechanisms, thereby further exacerbating mitochondrial dysfunction [29,30,31].

The differential ROS-scavenging capacities observed among the tested compounds may be attributed to their structural variations, particularly hydroxyl substitution and methylation patterns. The potent reducing properties conferred by hydroxyl groups likely enable direct ROS neutralization, thereby providing protection to the mitochondrial [38,39]. This aligns with reports that eremophilane-type sesquiterpenes reduce ROS and apoptosis in TGF-*β*_1_-induced BEAS-2B cells [40] and inhibit ROS production in activated neutrophils [41]. Molecular docking analyses by Gao et al. demonstrated strong binding interactions of phenylethanol glycosides with key molecular targets, including GSTP1, EGFR, and MAPK8 [42]. Qi et al. reported their neuroprotective effects against H_2_O_2_-induced oxidative stress and apoptosis through NOX_2_/ROS/MAPK pathway modulation [43], suggesting potential activation of endogenous antioxidant defense systems by these compounds, although their precise mechanisms require further investigation.

The experimental results demonstrated that all seven compounds conferred dose-dependent protection against LPS-induced mitochondrial damage in endothelial cells through multiple pathways. Notably, remophilanetriol K (**2**) and acteoside (**3**) exhibited significant protective effects against LPS-induced mitochondrial injury in endothelial cells by effectively suppressing ROS accumulation and preserving mitochondrial function through multiple mechanisms. These findings suggest their potential as molecular targets for treating sepsis-associated vascular mitochondrial dysfunction, although the detailed protective mechanisms require further investigation.

## 3. Materials and Methods

### 3.1. Experimental Protocols

NMR spectra were recorded on a Bruker AVANCE III 500 MHz spectrometer using CDCl_3_ as the reference (*δ*_H_ 7.26/*δ*_C_ 77.2). Semi-preparative HPLC was performed using a YMC ODS-A column (5 μm, 250 × 10 mm, YMC, Kyoto, Japan) with a SEP LC-52 system. UV (UV-2102, Unico, Shanghai, China) and IR (FTIR-8400S, Shimadzu, Kyoto, Japan) spectra were obtained. Optical rotations and CD spectra were measured using a 241 polarimeter (PerkinElmer, Waltham, MA, USA) and J-815 spectropolarimeter (JASCO, Tokyo, Japan), respectively. HR-ESI-MS data were acquired using a Xevo G2-S QTOF system (Waters, Milford, CT, USA).

### 3.2. UPLC-Q-TOF-MS/MS Parameters

For the analysis of mass spectrometry fragmentation behavior, compounds **7**–**10** were dissolved in methanol. The analysis was performed using a Waters Acquity UPLC-PDA system (Waters, Milford, CT, USA) equipped with a BEH C18 column (2.1 × 100 mm, 1.7 μm, Acquity BEH, Waters). The mobile phase comprised 0.1% formic acid/water (A) and 0.1% formic acid/acetonitrile (B) with the following gradient: 5% B (0–1 min), 5–95% B (1–13 min), 95% B (13–15 min), 95–5% B (15–16 min), and 5% B (16–18 min) at 0.3 mL/min. The UV absorbance (200–400 nm) and column temperature (40 °C) were set. For MS/MS analysis, full MS scans were conducted in positive ion mode from *m/z* 50 to 1500, with leucine-enkephalin ([M + H]^+^ = 556.2771, 200 ng/mL) as the lock mass. The ion source was maintained at 100 °C with desolvation at 450 °C (gas flow: 900 L/h), using 3.0 kV capillary and 40 V cone voltages. Low-energy scans used a collision energy of 4.0 eV, while high-energy scans were ramped from 20 to 40 eV. MassLynx 4.1 software was used to control the instruments.

### 3.3. Plant Material

In March 2024, fresh *R. glutinosa* roots were harvested in Wuzhi County (Jiaozuo City, Henan Province, China). Dr. Gang Ding from the Institute of Medicinal Plant Development identified the species. Triple EtOAc extraction of the root tuber phloem, followed by solvent evaporation, yielded 30.6 g of extract.

### 3.4. Purification of Compounds

The EtOAc extract (30.6 g) was fractionated using silica gel CC (100–200 mesh) with a CH_2_Cl_2_-MeOH gradient (1:0–0:1) to afford 15 fractions (Fr. 1–15). Through C-18 chromatography (MeOH/H_2_O, 1:9−1:0), sixteen fractions (Fr. 4.1–Fr. 4.16) were obtained from Fr. 4 (3.6 g). Fr. 4.10 (6.9 mg) was separated by semi-preparative HPLC (MeOH/H_2_O, 67–69% for 30 min and eluting in 69% for 15 min) to obtain compound **2** (1.2 mg, *t*_R_ = 25.6 min) and **1** (2.0 mg, *t*_R_ = 32.5 min). Fr. 12 (5.7 g) was eluted with MeOH in H_2_O (10−100%) from ODS chromatography to obtain two fractions (Fr. 12.1–Fr. 12.2). Under the HPLC conditions with MeOH in H_2_O 35–43% for 25 min and followed by 43% for 20 min, Fr. 12.1 (232.9 mg) was purified to obtain **5** (3.9 mg, *t*_R_ = 14.3 min), **6** (7.4 mg, *t*_R_ = 19.5 min), **3** (7.5 mg, *t*_R_ = 28.4 min), and **7** (11.3 mg, *t*_R_ = 29.8 min). Fr. 15 (642.5 mg) was loaded onto a Sephadex LH-20 column (MeOH) to afford five separations (Fr. 15.1–Fr. 15.5). And then Fr. 15.5 (75.6 mg) was eluted with 47% MeOH in H_2_O for 50 min by semi-preparative HPLC to yield **4** (25.1 mg, *t*_R_ = 26.1 min).

Remophilanetriol J (**1**): [α${]}_{D}^{25}$ −4.0 (*c* 0.05, MeOH); UV (MeOH) *λ*_max_ (log *ε*) 266 (3.79) nm; IR (neat) *ν*_max_ 3363, 2925, 2854, 1665, 1590, 1377, 1229, and 1039 cm^−1^; ^1^H NMR (500 MHz, CDCl_3_) and ^13^C NMR (125 MHz, CDCl_3_), shown in Table 1; (+)-HR-ESI-MS: *m/z* 235.1708 [M+H]^+^ (calcd. for C_15_H_23_O_2_, 235.1698).

Remophilanetriol K (**2**): [α${]}_{D}^{25}$ −15.0 (*c* 0.1, MeOH); UV (MeOH) *λ*_max_ (log *ε*) 237 (4.56) nm; IR (neat) *ν*_max_ 3356, 2937, 1667, 1387, and 1031 cm^−1^; ^1^H NMR (500 MHz, CDCl_3_) and ^13^C NMR (125 MHz, CDCl_3_), shown in Table 1; (+)-HR-ESI-MS: *m/z* 251.1645 [M + H]^+^ (calcd. for C_15_H_23_O_3_, 251.1647).

### 3.5. ECD Calculations

MMFF94S-based conformational sampling was performed, followed by DFT optimization of conformers within 10 kcal/mol of the global minimum. The benchmark results demonstrate that the dispersion-corrected functional B3LYP-D3BJ exhibits high accuracy, suggesting that it is suitable for biochemically relevant systems. Conformers within 0–10 kcal/mol were optimized at the B3LYP-D3BJ/6-31G (d) level, and those within 0–4 kcal/mol were reoptimized at the B3LYP-D3BJ/6-311G (2d, p) level. ECD calculations were conducted using TDDFT/B3LYP/6-311G (2d, p)/SMD. All computations were performed using Gaussian 09 software.

### 3.6. HUVEC Cultivation and Compound Treatment

HUVECs (ZQXZBIO, Shanghai, China) were maintained in Endothelial Cell Medium (Catalog #1001, ScienCell, Carlsbad, CA, USA) containing 100 U/mL penicillin–streptomycin at 37 °C/5% CO_2_. For cytotoxicity evaluation, cells were treated with graded drug concentrations (0, 1.5625, 3.125, 6.25, 12.5, 25, 50, 100, and 200 μM; 48 h) and analyzed by CCK-8 assay. To induce endothelial injury, HUVECs were stimulated with LPS (100 ng/mL, 48 h). For drug intervention studies, cells were pretreated with low/medium/high drug doses (12 h) prior to co-exposure to LPS (48 h). DMSO (0.1%) was used to improve the drug’s solubility and bioavailability.

### 3.7. CCK-8 Assay

HUVEC proliferation was assessed by CCK-8 assay (SparkJade, Jinan, China, CT0001-D). Cells (5 × 10^3^/well) were seeded in 96-well plates, treated, and incubated with CCK-8 reagent (10% in serum/antibiotic-free medium) (4 h, 37 °C). Absorbance at 450 nm was measured in triplicates using a microplate reader.

### 3.8. Transwell Assay

Treated HUVECs were resuspended in serum-free ECM and seeded into the upper chambers of the Transwell, while complete ECM was added to the lower chambers. After 6 h of migration, the cells were washed, fixed (20 min), and stained with H&E (hematoxylin: 10 min; eosin: 5 min). Migrated cells were quantified by counting the number of cells in multiple fields.

### 3.9. ROS Detection

Intracellular ROS levels were quantified using an assay kit (Beyotime, Shanghai, China, S0033S) following the manufacturer’s protocol. Cells subjected to different treatments were stained with 10 μM HDCFDA and incubated (20 min, 37 °C).

### 3.10. Mitotracker Staining

HUVECs were stained with 500 nmol/L MitoTracker Red CMXRos solution (9082, Cell Signaling Technology, Danvers, MA, USA) for 30 min. Images were acquired using a Leica M205FA stereofluorescence microscope. ImageJ 1 software was used to assess mitochondrial fluorescence intensity.

### 3.11. JC-1 Mitochondrial Membrane Potential Staining

Treated HUVECs were resuspended in serum-free ECM medium, allowed to adhere, and stained with 2 μM JC-1 (C2006, Beyotime) (20 min, 37 °C, dark). Following two washes with JC-1 buffer, cells were imaged using fluorescence microscopy in 1 mL culture medium.

### 3.12. Western Blot

HUVECs were lysed in RIPA buffer (Beyotime, P0013C) containing protease/phosphatase inhibitors. Protein concentration was determined using a BCA kit (Beyotime, P0012). After separation by 10% SDS-PAGE, the proteins were transferred to PVDF membranes (Millipore, Burlington, MA, USA, R1DB92457). Membranes were probed with primary antibodies against TOM20 (Proteintech, Chicago, IL, USA, 11802-1-AP, 1:1000) and DRP1 (CST, Danvers, MA, USA, 8570S, 1:1000) at 4 °C overnight, followed by HRP-conjugated secondary antibody (SparkJade, Jinan, China, EF0014, 1:10,000) for 1 h at RT. GAPDH (1:10,000, ZB15004-HRP-100, Servicebio, Wuhan, China) was used as the loading control, without the need for additional secondary antibody incubation. Protein bands were visualized using an Ultra-Sensitive Chemiluminescent Substrate Kit (ED0015-B, SparkJade, Jinan, China) and quantified using ImageJ 1 software.

## 4. Conclusions

This study reports the isolation and characterization of two novel eremophilane-type sesquiterpenes, remophilanetriol J (**1**) and remophilanetriol K (**2**), along with five known phenylethanol glycosides (**3**–**7**), from the fresh roots of *R. glutinosa*. Additionally, the diagnostic mass spectrometric fragmentation pathways for phenylethanol glycosides (**3**–**7**) were elucidated for the first time, establishing a reliable analytical method for identifying analogous compounds in complex botanical extracts.

The protective effects of all seven isolated compounds (**1**–**7**) against LPS-induced damage in HUVECs were investigated, revealing significant dose-dependent cytoprotective activity. Treatment with these compounds reduced intracellular ROS levels, ameliorated mitochondrial membrane potential depolarization, alleviated TOM20 expression suppression, and inhibited DRP1 overexpression, collectively attenuating mitochondrial dysfunction and endothelial cell apoptosis. Notably, remophilanetriol K (**2**) exhibited potent protective effects at a low concentration (1.5625 μM), significantly improving LPS-induced endothelial cell viability (80.00 ± 6.93%) compared to the LPS model group. This compound effectively reduced intracellular ROS levels (74.78 ± 4.56%), mitigated mitochondrial membrane potential depolarization (75.36 ± 10.82%), restored TOM20 expression (62.25 ± 9.69%), and suppressed DRP1 overexpression (81.81 ± 5.68%), demonstrating strong protection against LPS-induced mitochondrial damage in vascular endothelial cells. These findings provide a mechanistic foundation for the traditional use of *R. glutinosa* and offer valuable insights into the development of novel therapeutic agents targeting mitochondrial dysfunction in sepsis.

## Figures and Tables

**Table 1:** ^1^H and ^13^C NMR data of compounds **1** and **2** in CDCl_3_.

Position	1		2	
	*δ*H ^a^ (mult, *J* in Hz)	*δ*C ^b^, Type	*δ*H ^a^ (mult, *J* in Hz)	*δ*C ^b^, Type
1a	1.29, overlapped	20.7, CH_2_	1.32, dd, (11.5, 13.0)	30.1, CH_2_
1b	2.00, ddd, (11.5, 3.0, 1.5)		2.31, ddd, (13.0, 4.5, 2.5)	
2a	1.30, overlapped	25.3, CH_2_	3.70, tt, (11.5, 4.5)	69.9, CH
2b	1.83, m			
3a	1.25, overlapped	30.2, CH_2_	1.28, td, (13.0, 11.5)	39.1, CH_2_
3b	1.44, ddd, (13.0, 5.0, 2.5)		1.78, ddd, (13.0, 4.5, 2.5)	
4	1.55, m	43.3, CH	1.63, overlapped	41.4, CH
5		40.0, C		38.9, C
6a	2.30, dd, (2.5, 17.5)	41.1, CH_2_	2.27, overlapped	40.7, CH_2_
6b	2.68, d, (17.5)		2.70, d, (17.5)	
7		151.8, C		151.9, C
8	6.05, d, (2.5)	124.0, CH	6.08, d, (2.5)	123.6, CH
9		201.9, C		200.2, C
10	2.21, dd, (3.5, 11.5)	55.2, CH	2.26, overlapped	53.1, CH
11		146.4, C		145.9, C
12	5.58, s 5.60, s	117.3, CH_2_	5.60, s 5.61, s	117.5, CH_2_
13	4.38, s	63.8, CH_2_	4.39, s	63.4, CH_2_
14	0.77, s	11.9, CH_3_	0.78, s	11.7, CH_3_
15	0.90, d, (7.0)	15.4, CH_3_	0.96, d, (7.0)	14.9, CH_3_

^a^ Recorded at 500 MHz, ^b^ Recorded at 125 MHz.

## Data Availability

The original contributions presented in this study are included in the article/Supplementary Material. Further inquiries can be directed to the corresponding authors.

## Supplementary Materials

The following supporting information can be downloaded at https://www.mdpi.com/article/10.3390/ph18081125/s1, Figure S1: ^1^H NMR spectrum (500 MHz) of compound **1** in CDCl_3_. Figure S2: ^13^C NMR spectrum (125 MHz) of compound **1** in CDCl_3_. Figure S3: ^1^H-^1^H COSY spectrum (500 MHz) of compound **1** in CDCl_3_. Figure S4: HSQC spectrum (500 MHz) of compound **1** in CDCl_3_. Figure S5: HMBC spectrum (500 MHz) of compound **1** in CDCl_3_. Figure S6: NOE spectrum (500 MHz) of compound **1** in CDCl_3_. Figure S7: UPLC-Q-TOF-MS/MS spectra of compound **1** in CH_3_OH. Figure S8: IR spectrum of compound **1**. Figure S9: UV spectrum of compound **2** in CH_3_OH. Figure S10: ^1^H NMR spectrum (500 MHz) of compound **2** in CDCl_3_. Figure S11: ^13^C NMR spectrum (125 MHz) of compound **2** in CDCl_3_. Figure S12: ^1^H-^1^H COSY spectrum (500 MHz) of compound **2** in CDCl_3_. Figure S13: HSQC spectrum (500 MHz) of compound **2** in CDCl_3_. Figure S14: HMBC spectrum (500 MHz) of compound **2** in CDCl_3_. Figure S15: UPLC-Q-TOF-MS/MS spectra of compound **2** in CH_3_OH. Figure S16: IR spectrum of compound **2**. Figure S17: UV spectrum of compound **2** in CH_3_OH. Figure S18: UPLC-Q-TOF-MS/MS spectra of compound **3** in CH_3_OH. Figure S19: UPLC-Q-TOF-MS/MS spectra of compound **4** in CH_3_OH. Figure S20: UPLC-Q-TOF-MS/MS spectra of compound **5** in CH_3_OH. Figure S21: UPLC-Q-TOF-MS/MS spectra of compound **6** in CH_3_OH. Figure S22: UPLC-Q-TOF-MS/MS spectra of compound **7** in CH_3_OH. Figure S23: Possible mass fragmentation pathways of **3**. Figure S24: Possible mass fragmentation pathways of **4**. Figure S25: Possible mass fragmentation pathways of **6**. Figure S26: Possible mass fragmentation pathways of **7**. Table S1: Eremophilane-type sesquiterpenes isolated from *Rehmannia glutinosa*. Table S2: Elemental constituents of major ions from UPLC-Q-TOF-MS/MS spectra for acteoside (**3**). Table S3: Elemental constituents of major ions from UPLC-Q-TOF-MS/MS spectra for compound (**4**). Table S4: Elemental constituents of major ions from UPLC-Q-TOF-MS/MS spectra for compound (**5**). Table S5: Elemental constituents of major ions from UPLC-Q-TOF-MS/MS spectra for compound (**6**). Table S6: Elemental constituents of major ions from UPLC-Q-TOF-MS/MS spectra for compound (**7**). Table S7: IC_50_ values for cytotoxic effect of **1**–**7** on endothelial cells. Table S8: Experimental concentrations of compounds (**1**–**7**) (μM). Table S9: The extent to which compounds (**1**–**7**) alleviate LPS-induced impairment of HUVEC migration (*p* < 0.05). Table S10: Effects of compounds (**1**–**7**) on ROS levels in LPS-induced HUVECs (*p* < 0.05). Table S11: The extent to which compounds (**1**–**7**) restore LPS-induced reduction in mitochondrial fluorescence intensity in HUVECs (via MitoTracker staining) (*p* < 0.05). Table S12: The extent to which compounds (**1**–**7**) restore the LPS-induced reduction in mitochondrial red/green fluorescence intensity ratios in HUVECs (via JC-1 staining) (*p* < 0.05). Table S13: The extent to which compounds (**1**–**7**) restore the LPS-induced reduction in TOM20 protein expression in HUVECs (*p* < 0.05). Table S14: The extent to which compounds (**1**–**7**) reverse the LPS-induced upregulation of DRP1 protein expression in HUVECs (*p* < 0.05). References [15,18,40,44,45] are cited in the supplementary materials.

## Abbreviations

The following abbreviations are used in this manuscript:

IR	Infrared Radiation
UV	Ultraviolet
MS	Mass Spectrum
NMR	Nuclear Magnetic Resonance
LPS	Lipopolysaccharide
ECD	Electronic Circular Dichroism
HUVECs	Human Umbilical Vein Endothelial Cells
ROS	Reactive Oxygen Species
DFT	Density Functional Theory
ECM	Endothelial Cell Medium
