# Supplementary material for: Secondary Metabolites from Rehmannia glutinosa Protect Mitochondrial Function in LPS-Injured Endothelial Cells
Source: Pharmaceuticals (Basel). 2025 Jul 27;18(8):1125. doi: 10.3390/ph18081125 (PMC12389007; doi:10.3390/ph18081125)
Supplement: Supplementary file 1 [file pharmaceuticals-18-01125-s001.zip › pharmaceuticals-3762278-supplementary.pdf]

Supporting Information

Secondary Metabolites from *Rehmannia glutinosa* Protect Mitochondrial Function in LPS-Injured Endothelial Cells

Liwen Zhong[1,†], Mengkai Lu[2,†], Huiqi Fang[1], Chao Li[2,*], Hua Qu[3], Gang Ding[1,*]

[1] State Key Laboratory of Bioactive Substance and Function of Natural Medicines, Institute of Medicinal Plant Development, Chinese Academy of Medical Sciences and Peking Union Medical College, Beijing 100193, China.

2 College of Traditional Chinese Medicine, Shandong University of Traditional Chinese Medicine, Jinan 250355, China.

3 Xiyuan Hospital, China Academy of Chinese Medical Sciences, Beijing 100091, People's Republic of China.

* Correspondence: lichao71795@hotmail.com (L. Chao), hua_qu@yeah.net (H. Qu), gding@implad.ac.cn (G. Ding).

† These authors contributed equally to this work.

Table of Contents

Figure S1 ^{1}H NMR spectrum (500 MHz) of compound **1** in $CDCl_3$ 4

Figure S2 ^{13}C NMR spectrum (125 MHz) of compound **1** in $CDCl_3$ 4

Figure S3 ^{1}H-^{1}H COSY spectrum (500 MHz) of compound **1** in $CDCl_3$ 5

Figure S4 HSQC spectrum (500 MHz) of compound **1** in $CDCl_3$ 5

Figure S5 HMBC spectrum (500 MHz) of compound **1** in $CDCl_3$ 6

Figure S6 NOE spectrum (500 MHz) of compound **1** in CDCl3 6

Figure S7 UPLC-Q-TOF-MS/MS spectra of compound **1** in CH_3OH 7

Figure S8 IR spectrum of compound **1**....7

Figure S9 UV spectrum of compound **2** in CH_3OH 7

Figure S10 ^{1}H NMR spectrum (500 MHz) of compound **2** in $CDCl_3$ 8

Figure S11 ^{13}C NMR spectrum (125 MHz) of compound **2** in $CDCl_3$ 8

Figure S12 ^{1}H-^{1}H COSY spectrum (500 MHz) of compound **2** in $CDCl_3$ 9

Figure S13 HSQC spectrum (500 MHz) of compound **2** in $CDCl_3$ 9

Figure S14 HMBC spectrum (500 MHz) of compound **2** in $CDCl_3$ 10

Figure S15 UPLC-Q-TOF-MS/MS spectra of compound **2** in CH_3OH 10

Figure S16 IR spectrum of compound **2**....11

Figure S17 UV spectrum of compound **2** in CH_3OH 11

Figure S18 UPLC-Q-TOF-MS/MS spectra of compound **3** in CH_3OH. 11

Figure S19 UPLC-Q-TOF-MS/MS spectra of compound **4** in CH_3OH. 12

Figure S20 UPLC-Q-TOF-MS/MS spectra of compound **5** in CH_3OH. 12

Figure S21 UPLC-Q-TOF-MS/MS spectra of compound **6** in CH_3OH. 12

Figure S22 UPLC-Q-TOF-MS/MS spectra of compound **7** in CH_3OH. 12

Figure S23 Possible mass fragmentation pathways of **3.** 13

Figure S24 Possible mass fragmentation pathways of **4.** 14

Figure S25 Possible mass fragmentation pathways of **6.** 15

Figure S26 Possible mass fragmentation pathways of **7**. 16

Table S1 Eremophilane-type sesquiterpenes isolated from *Rehmannia glutinosa*....17

Table S2 Elemental constituents of major ions from UPLC-Q-TOF-MS/MS spectra for acteoside (**3**). 19

Table S3 Elemental constituents of major ions from UPLC-Q-TOF-MS/MS spectra for compound (**4**) 19

Table S4 Elemental constituents of major ions from UPLC-Q-TOF-MS/MS spectra for compound (**5**) 20

Table S5 Elemental constituents of major ions from UPLC-Q-TOF-MS/MS spectra for compound (**6**) 20

Table S6 Elemental constituents of major ions from UPLC-Q-TOF-MS/MS spectra for compound (**7**) 21

Table S7 IC_{50} values for cytotoxic effect of **1**–**7** on endothelial cells 21

Table S8 Experimental concentrations of compounds (**1**–**7**) (μM). 21

Table S9 The extent of compounds (**1**–**7**) alleviate LPS-induced impairment of HUVEC migration (p < 0.05).. **Error! Bookmark not defined.**

Table S10 Effects of compounds (**1**–**7**) on ROS levels in LPS-induced HUVECs (p < 0.05). 21

Table S11 The extent to which compounds (**1**–**7**) restore LPS-induced reduction in mitochondrial fluorescence intensity in HUVECs (via MitoTracker staining) (p < 0.05). 22

Table S12 The extent to which compounds (**1**–**7**) restore the LPS-induced reduction in mitochondrial red/green fluorescence intensity ratios in HUVECs (via JC-1 staining) (p < 0.05)..22

Table S13 The extent to which compounds (**1**–**7**) restore the LPS-induced reduction of TOM20 protein expression in HUVECs (p < 0.05)...22

Table S14 The extent to which compounds (**1**–**7**) reverse the LPS-induced upregulation of DRP1 protein expression in HUVECs (p < 0.05)..23

Figure S1 ^{1}H NMR spectrum (500 MHz) of compound **1** in $CDCl_3$

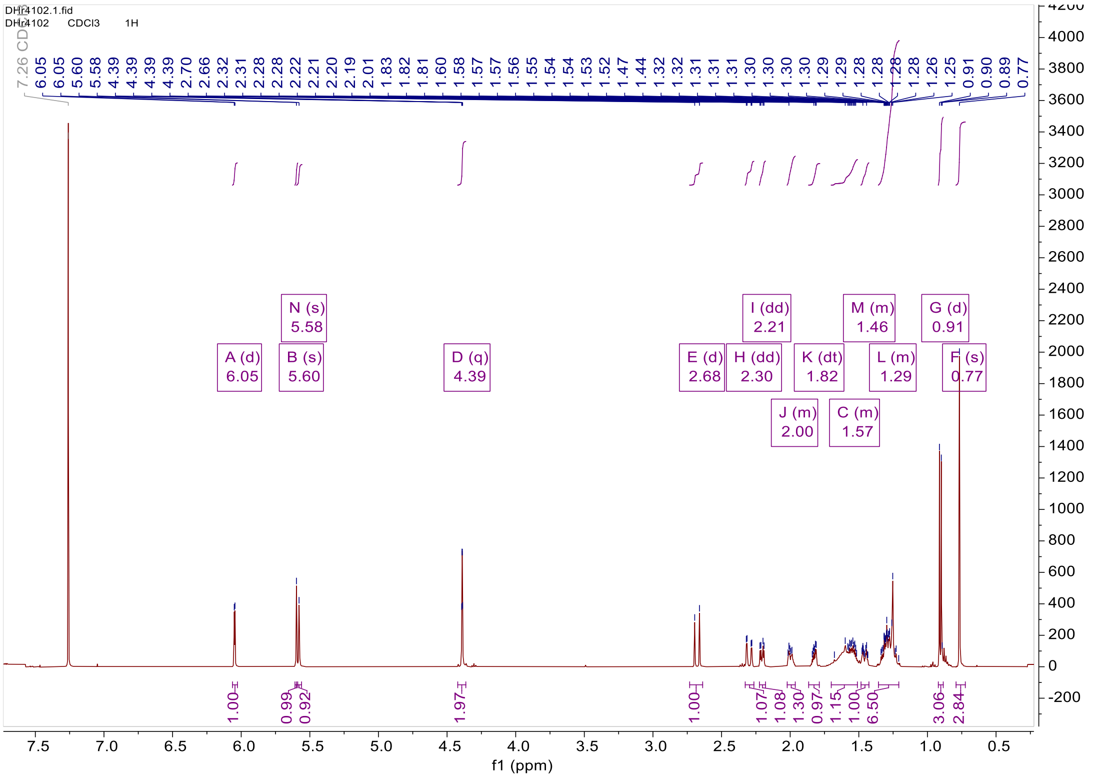

Figure S2 ^{13}C NMR spectrum (125 MHz) of compound **1** in $CDCl_3$

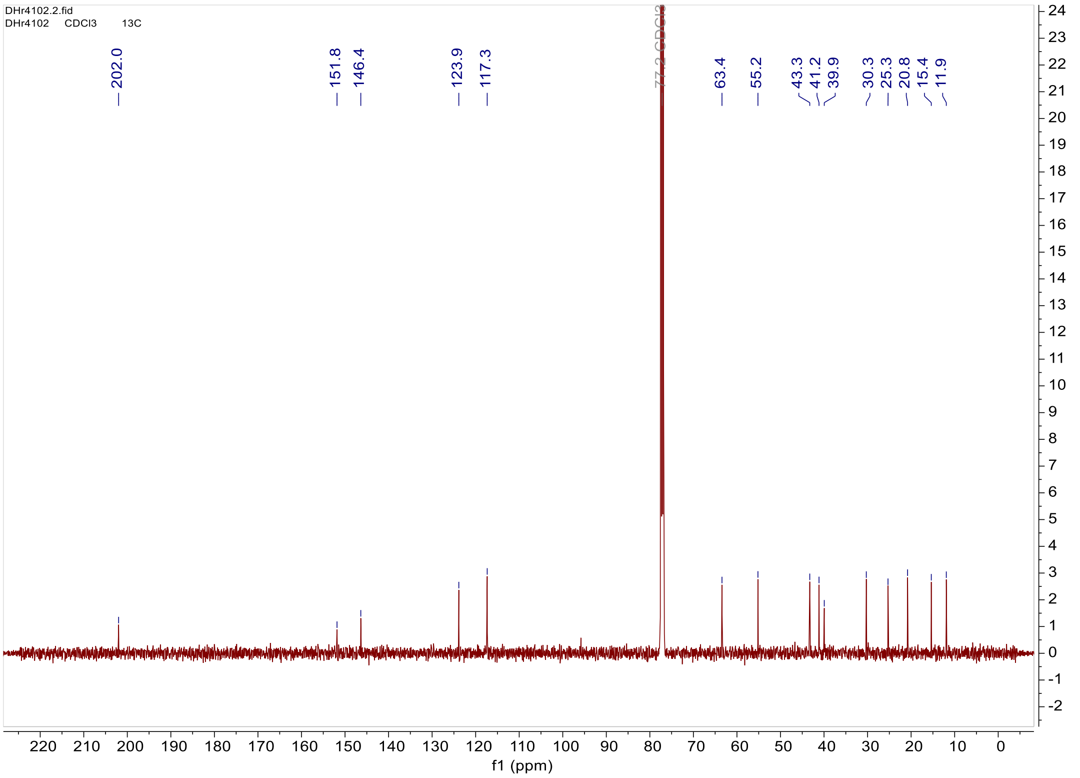

Figure S3 ^{1}H-^{1}H COSY spectrum (500 MHz) of compound **1** in $CDCl_3$

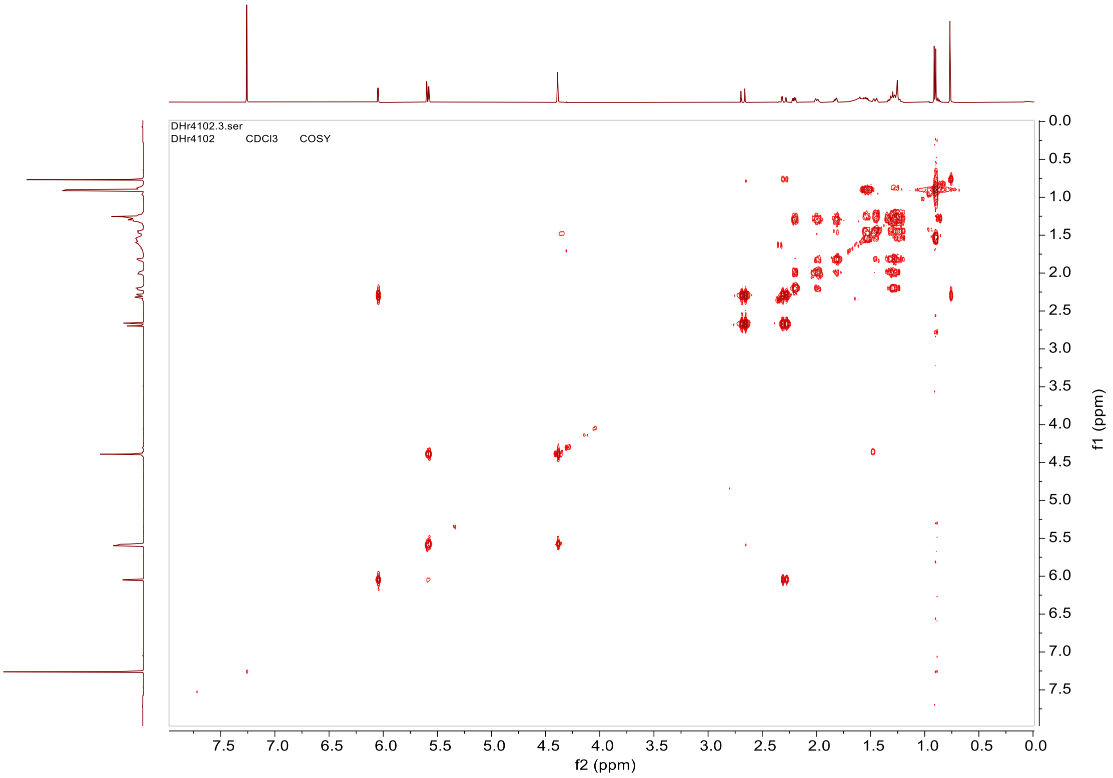

Figure S4 HSQC spectrum (500 MHz) of compound **1** in $CDCl_3$

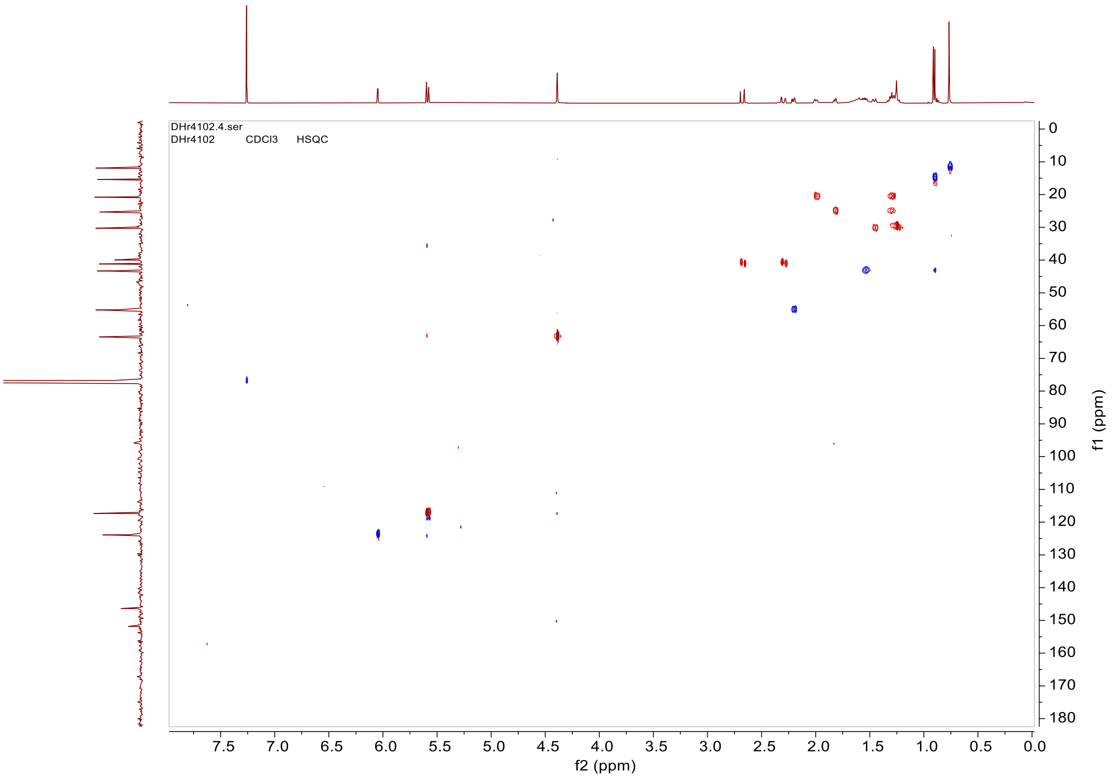

Figure S5 HMBC spectrum (500 MHz) of compound **1** in $CDCl_3$

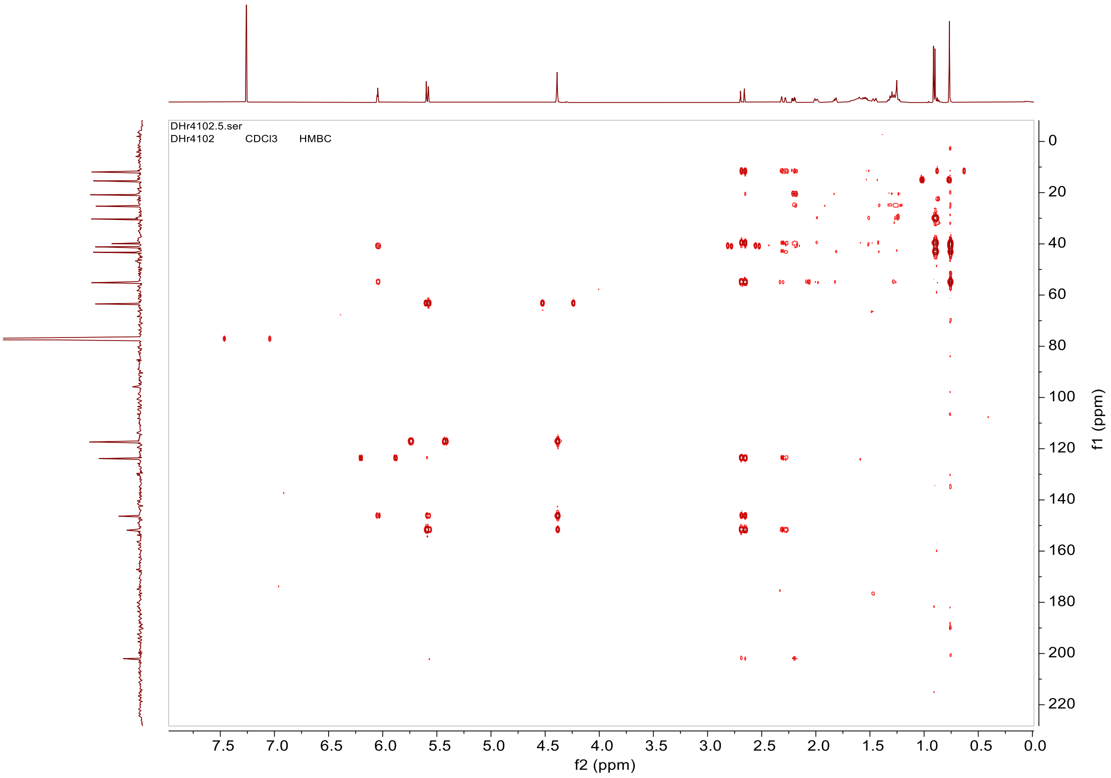

Figure S6 NOE spectrum (500 MHz) of compound **1** in CDCl3

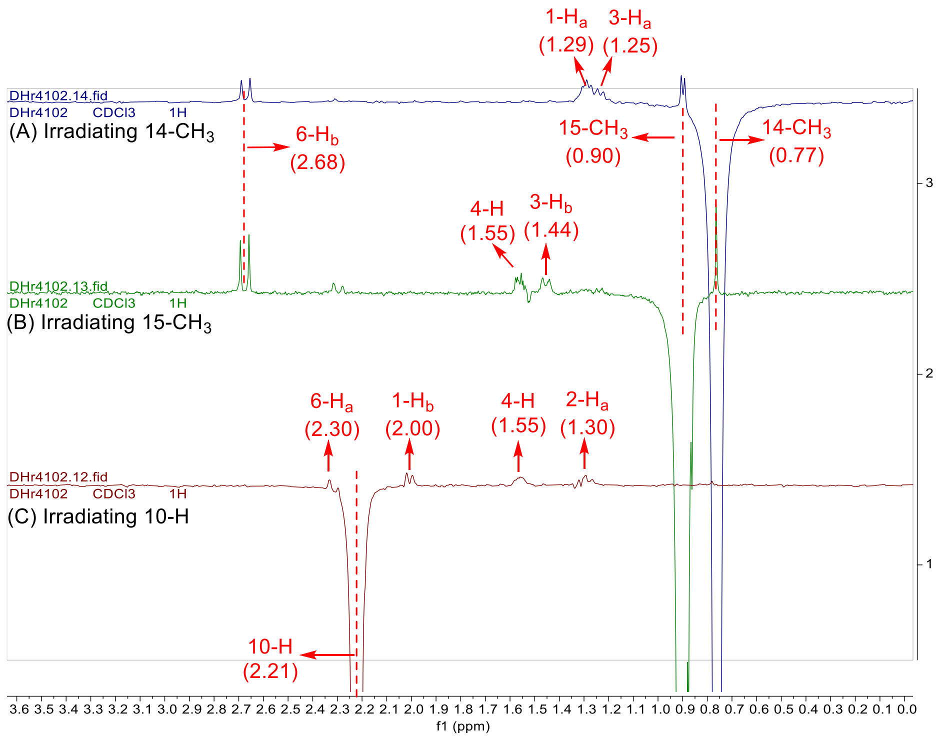

Figure S7 UPLC-Q-TOF-MS/MS spectra of compound **1** in CH_3OH

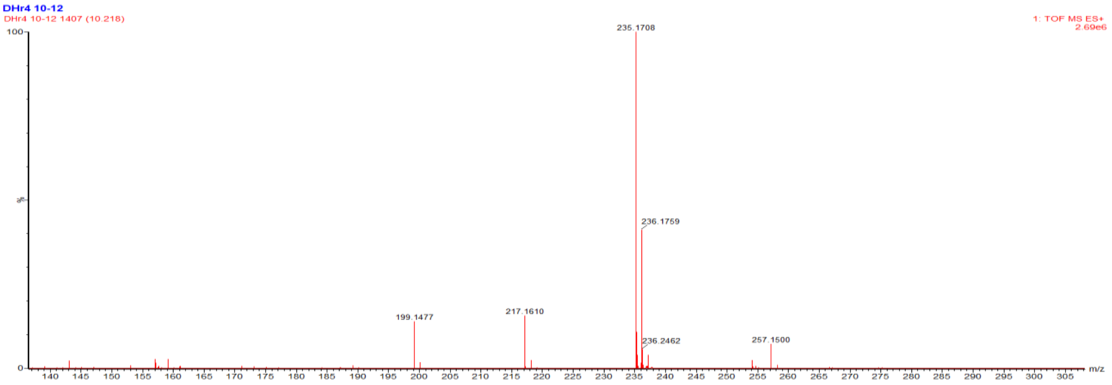

Figure S8 IR spectrum of compound **1**

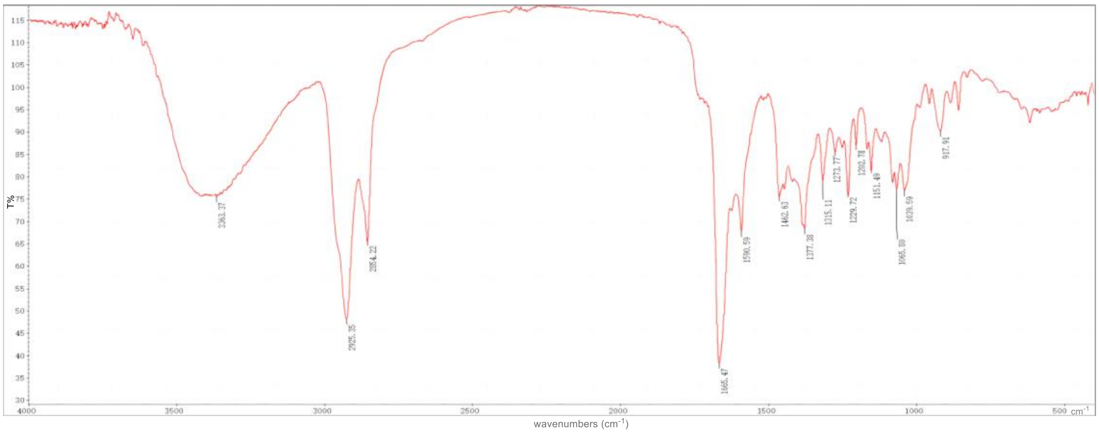

Figure S9 UV spectrum of compound **2** in CH_3OH

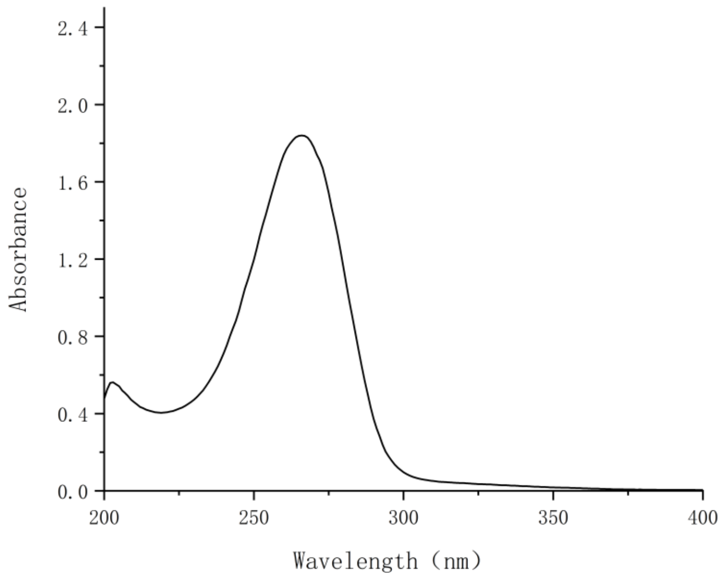

Figure S10 ^{1}H NMR spectrum (500 MHz) of compound **2** in $CDCl_3$

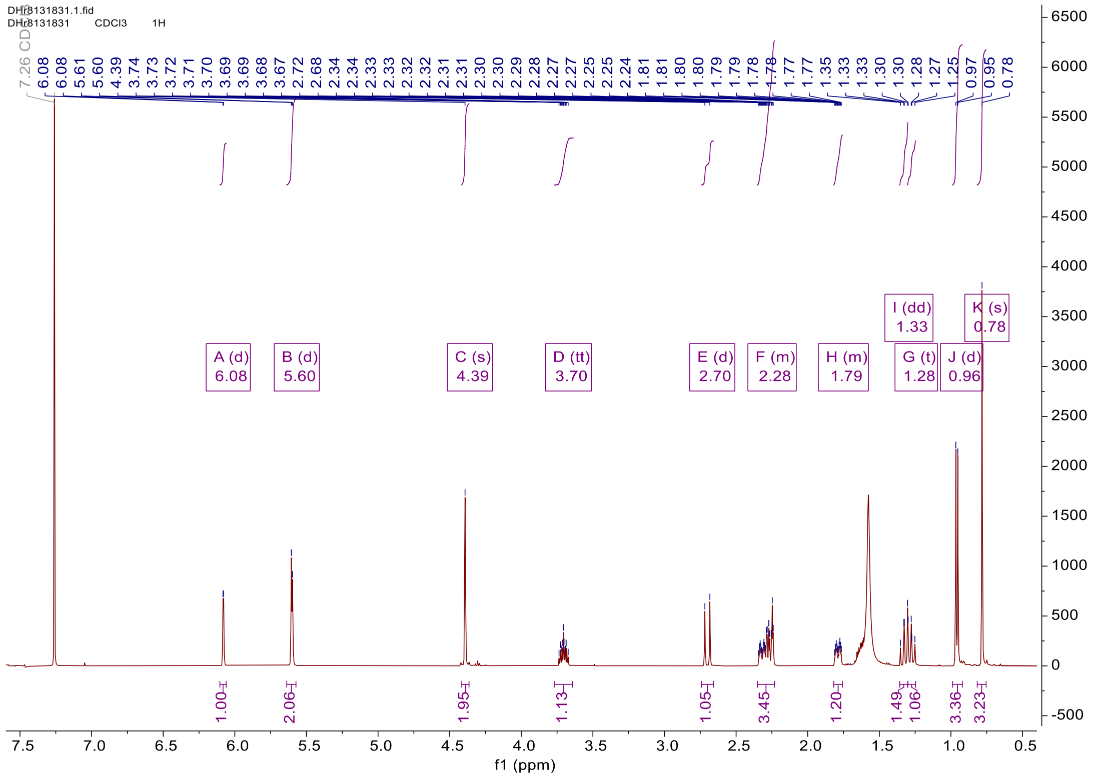

Figure S11 ^{13}C NMR spectrum (125 MHz) of compound **2** in $CDCl_3$

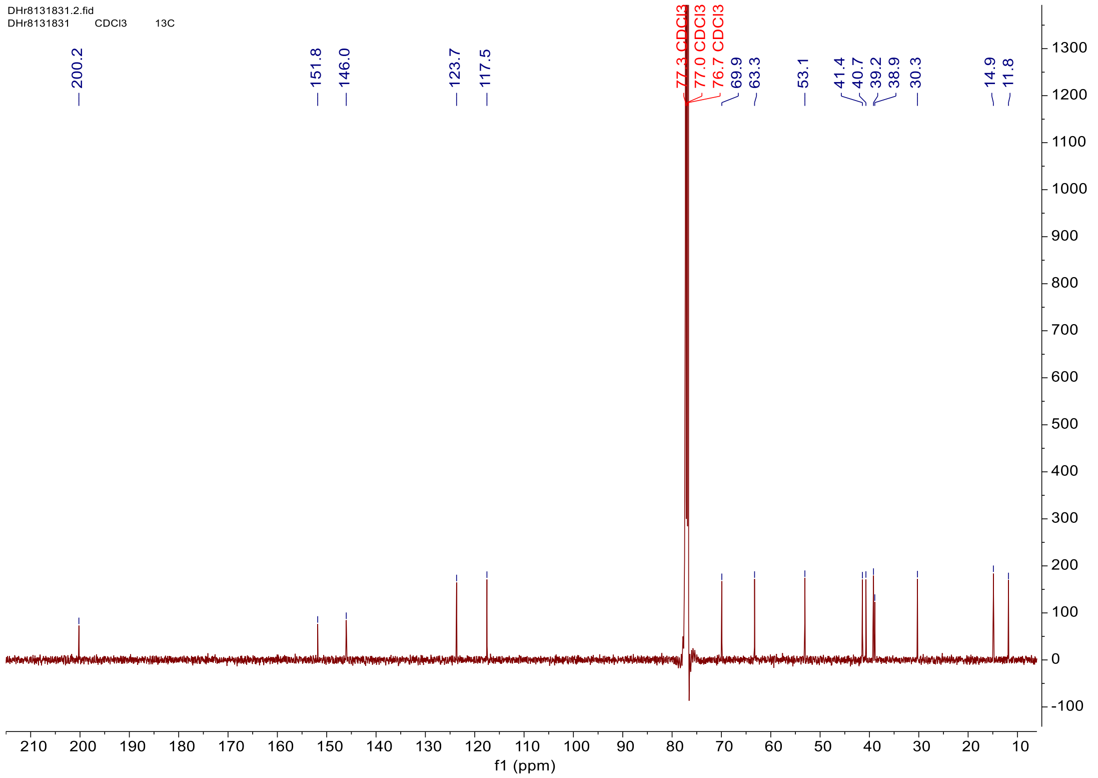

Figure S12 ^{1}H-^{1}H COSY spectrum (500 MHz) of compound **2** in $CDCl_3$

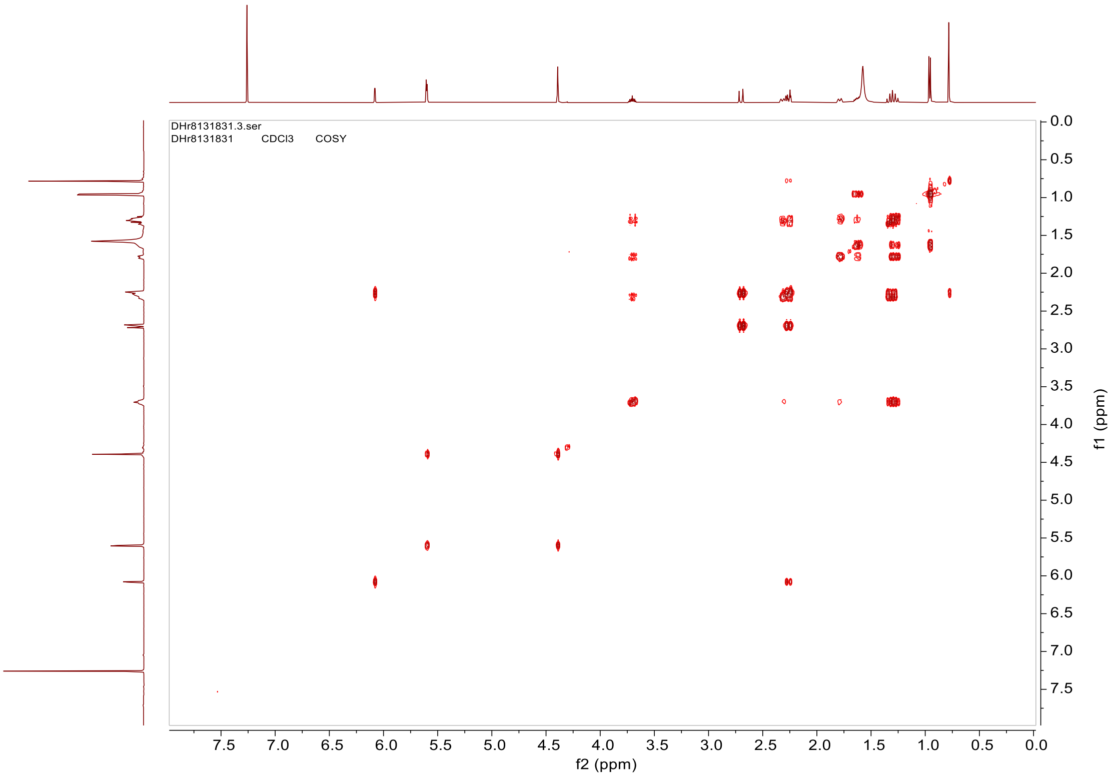

Figure S13 HSQC spectrum (500 MHz) of compound **2** in $CDCl_3$

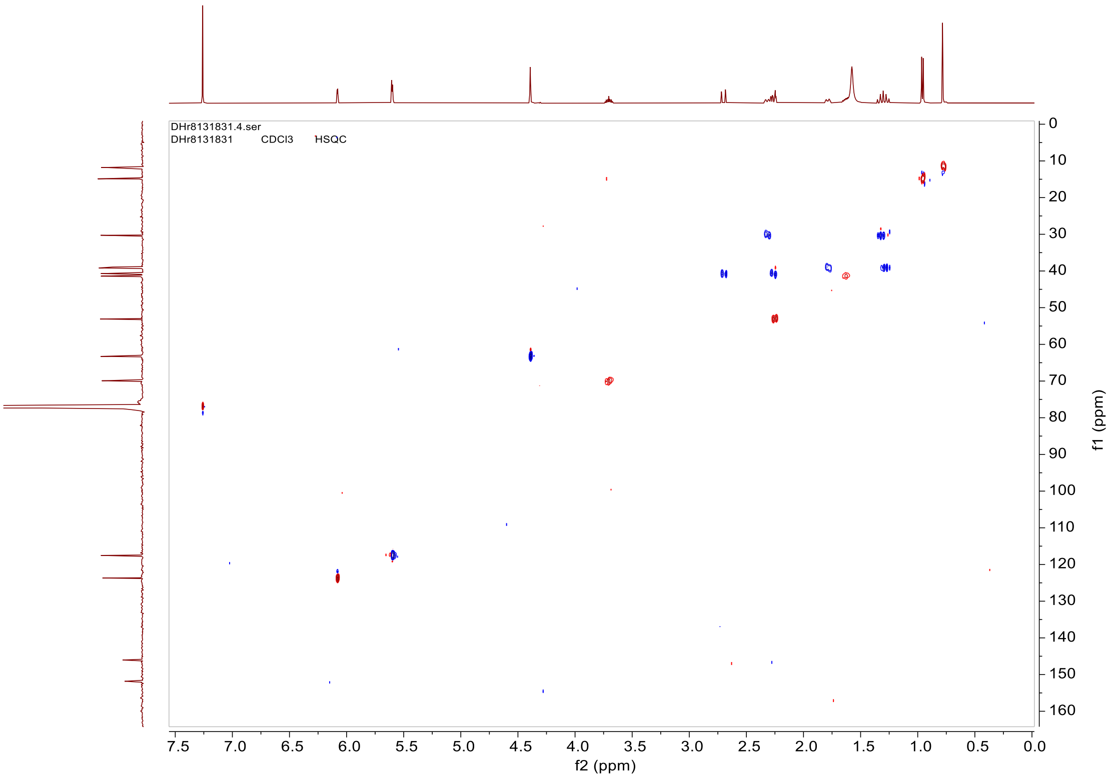

Figure S14 HMBC spectrum (500 MHz) of compound **2** in $CDCl_3$

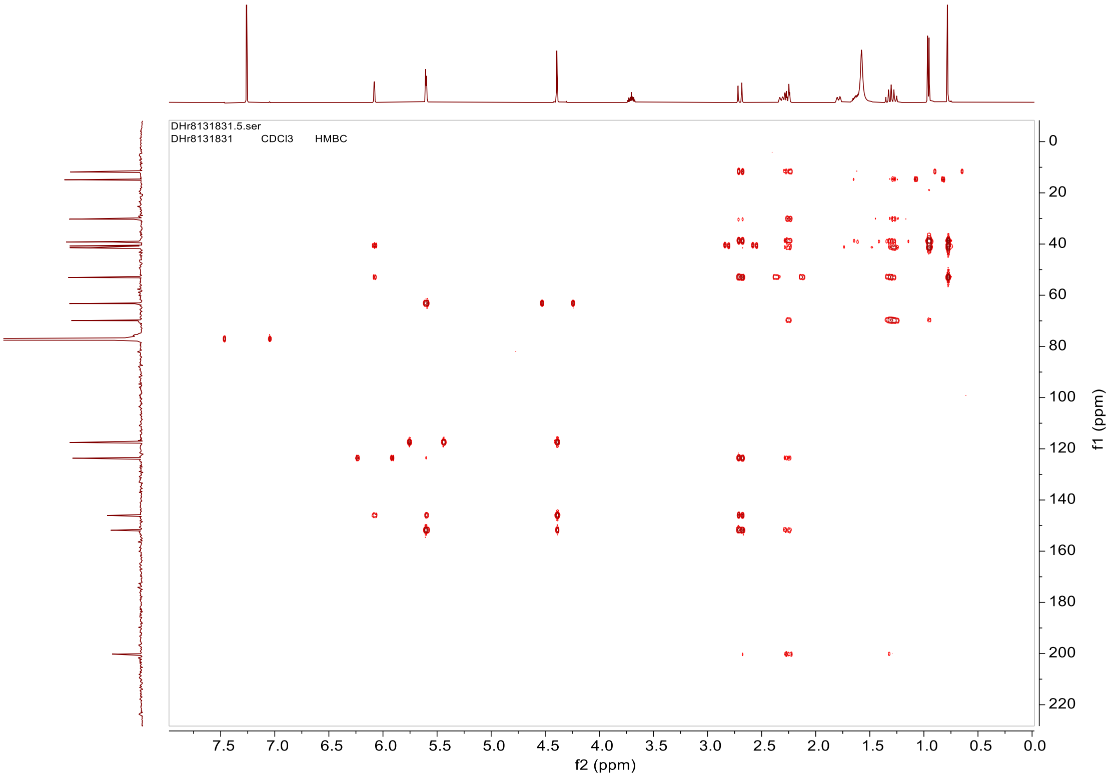

Figure S15 UPLC-Q-TOF-MS/MS spectra of compound **2** in CH_3OH

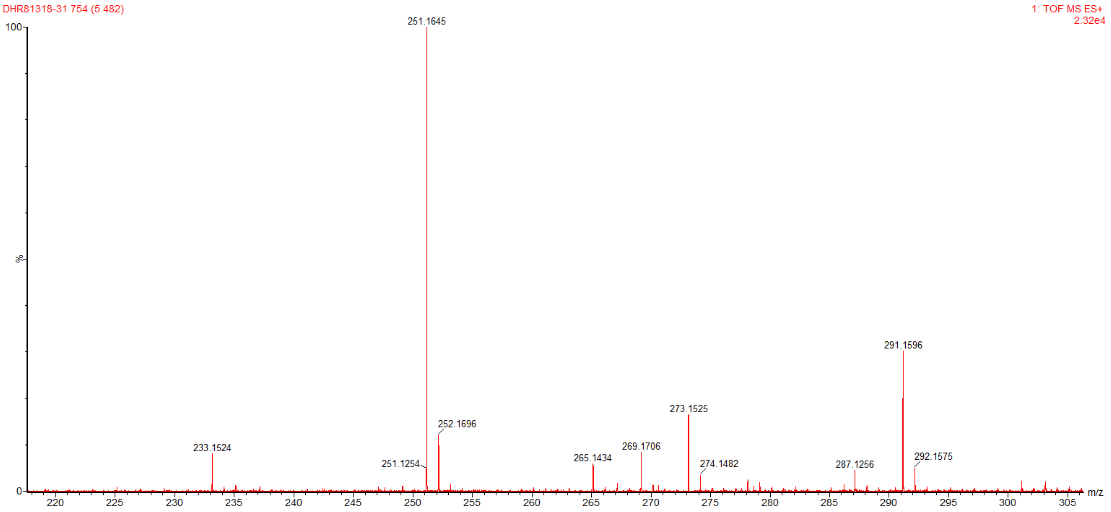

Figure S16 IR spectrum of compound **2**

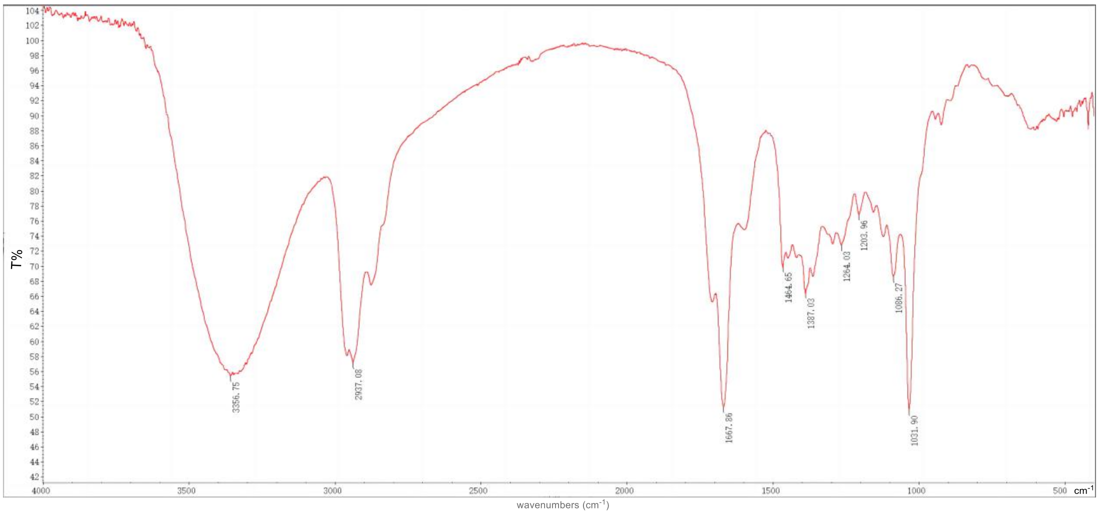

Figure S17 UV spectrum of compound **2** in CH_3OH

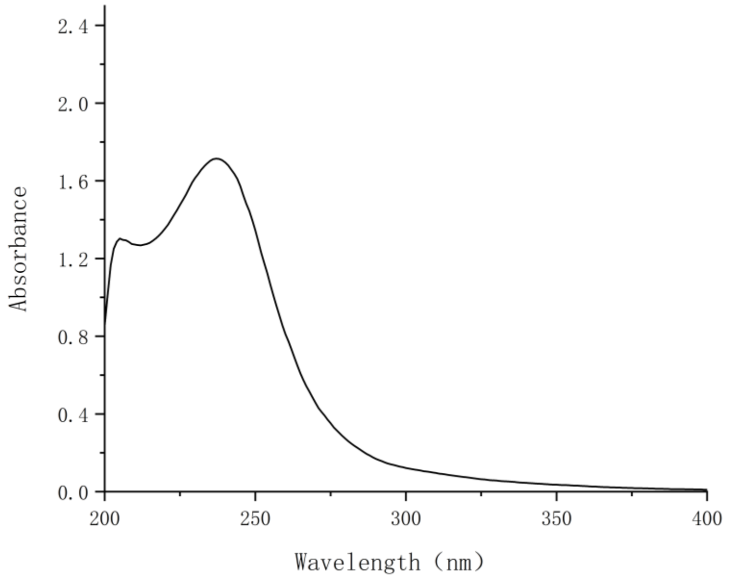

Figure S18 UPLC-Q-TOF-MS/MS spectra of compound **3** in CH_3OH.

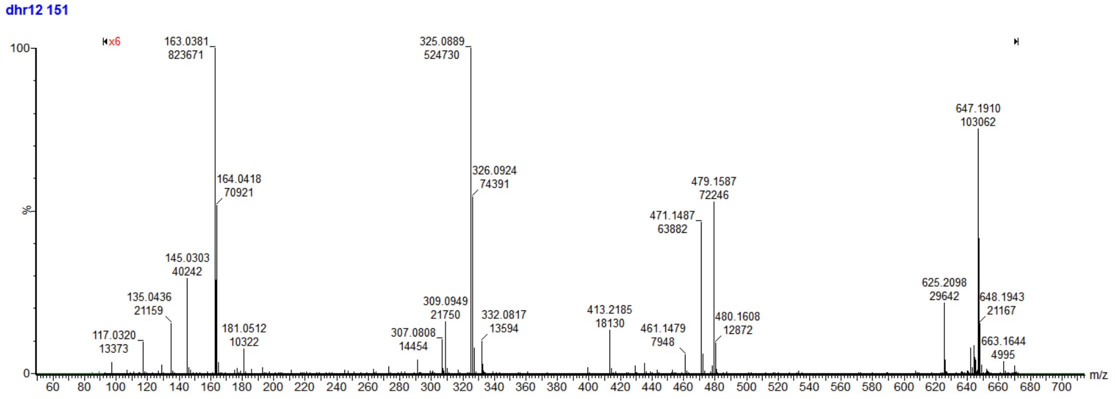

Figure S19 UPLC-Q-TOF-MS/MS spectra of compound **4** in CH_3OH.

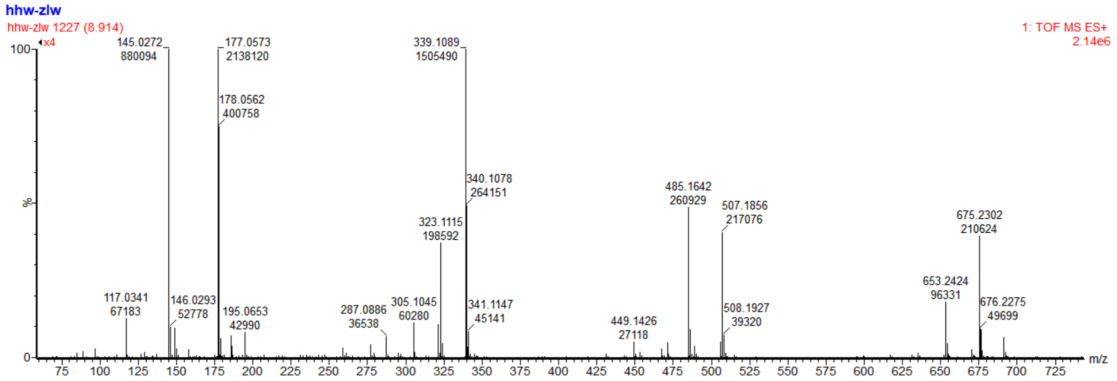

Figure S20 UPLC-Q-TOF-MS/MS spectra of compound **5** in CH_3OH.

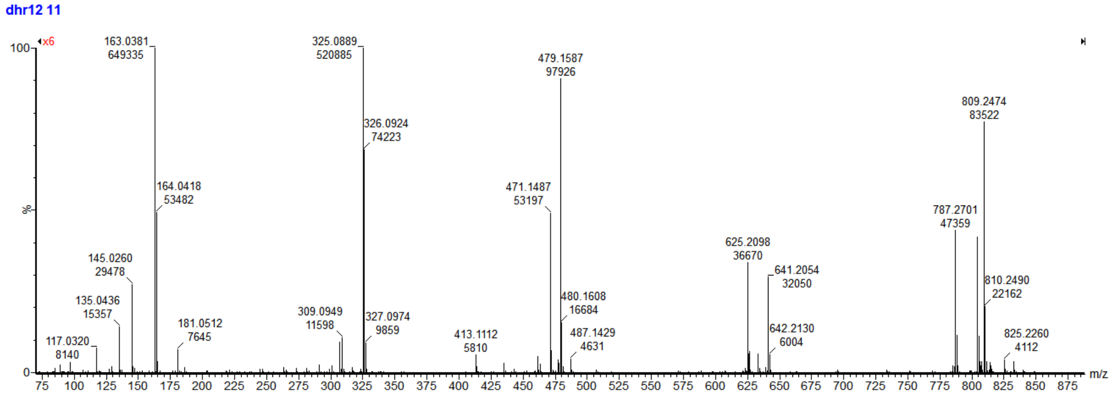

Figure S21 UPLC-Q-TOF-MS/MS spectra of compound **6** in CH_3OH.

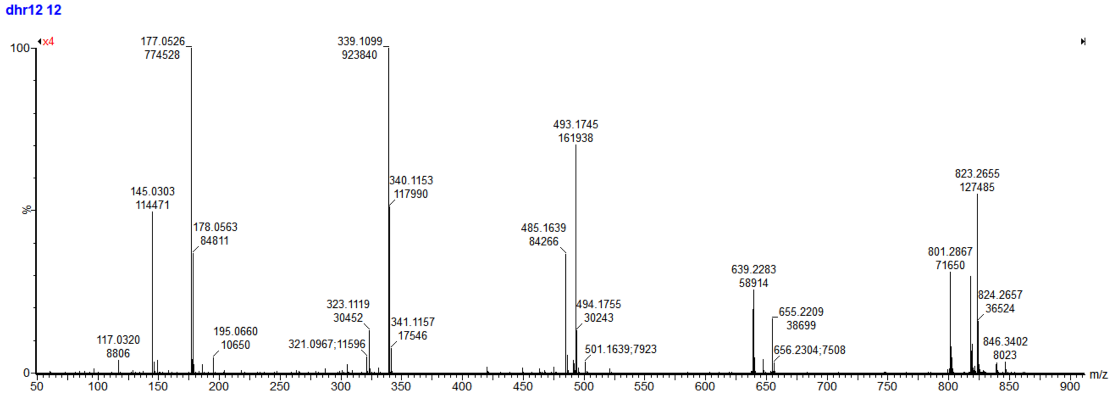

Figure S22 UPLC-Q-TOF-MS/MS spectra of compound **7** in CH_3OH.

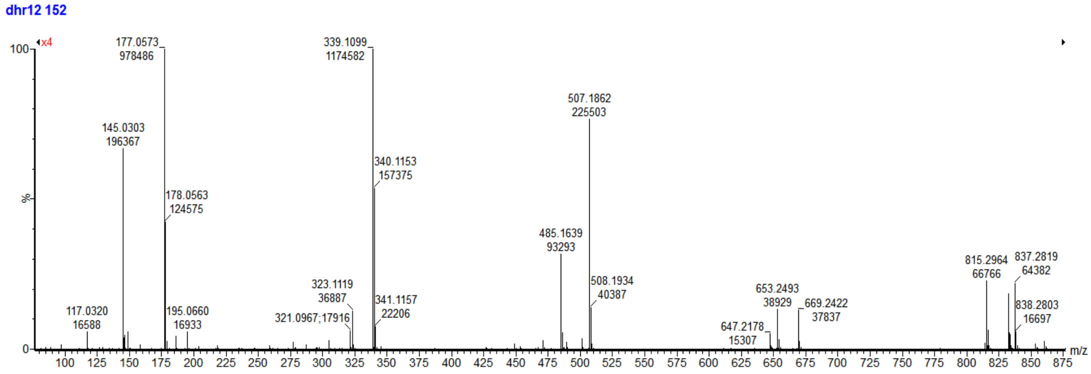

Figure S23 Possible mass fragmentation pathways of **3**.

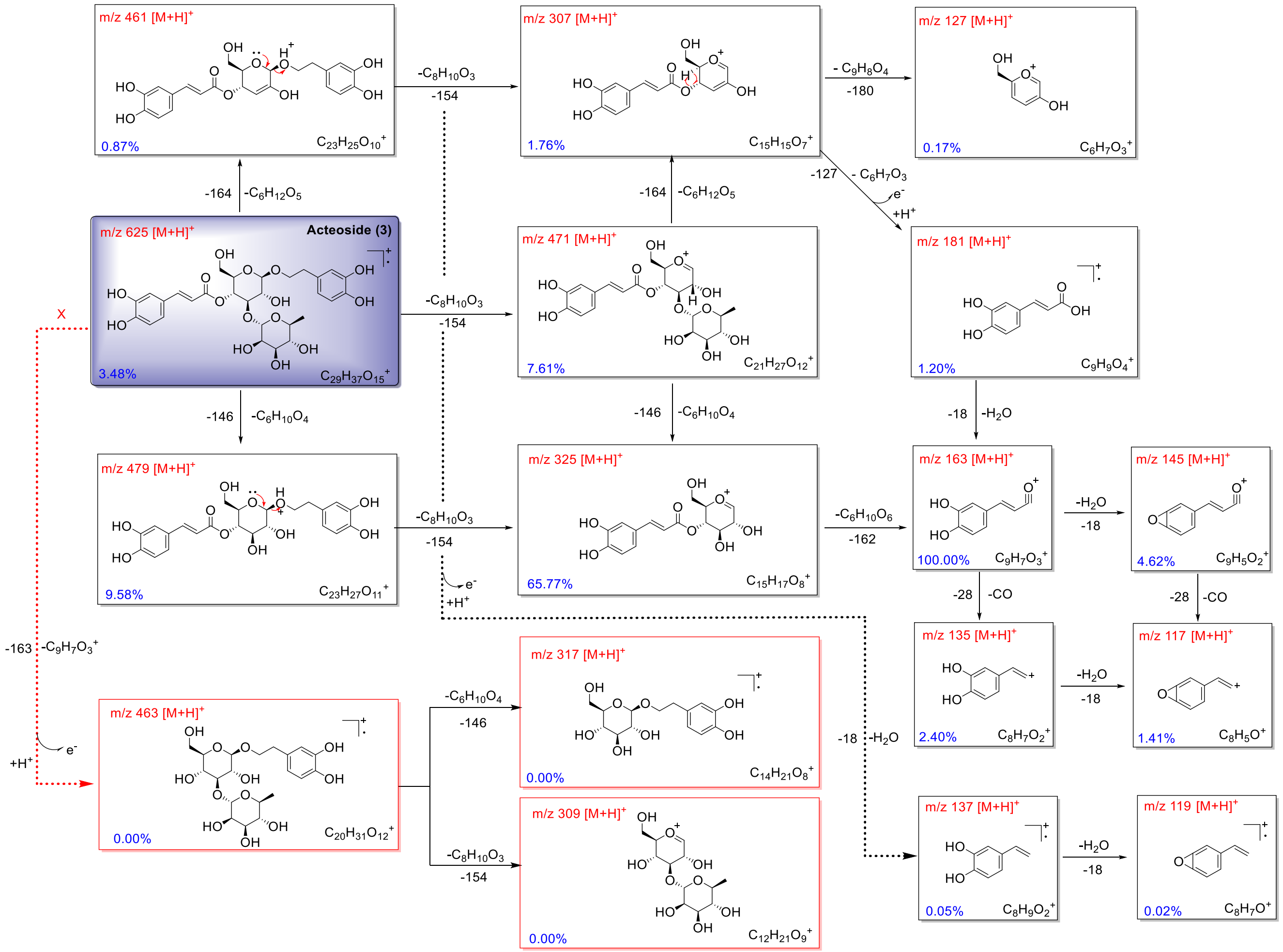

Figure S24 Possible mass fragmentation pathways of **4**.

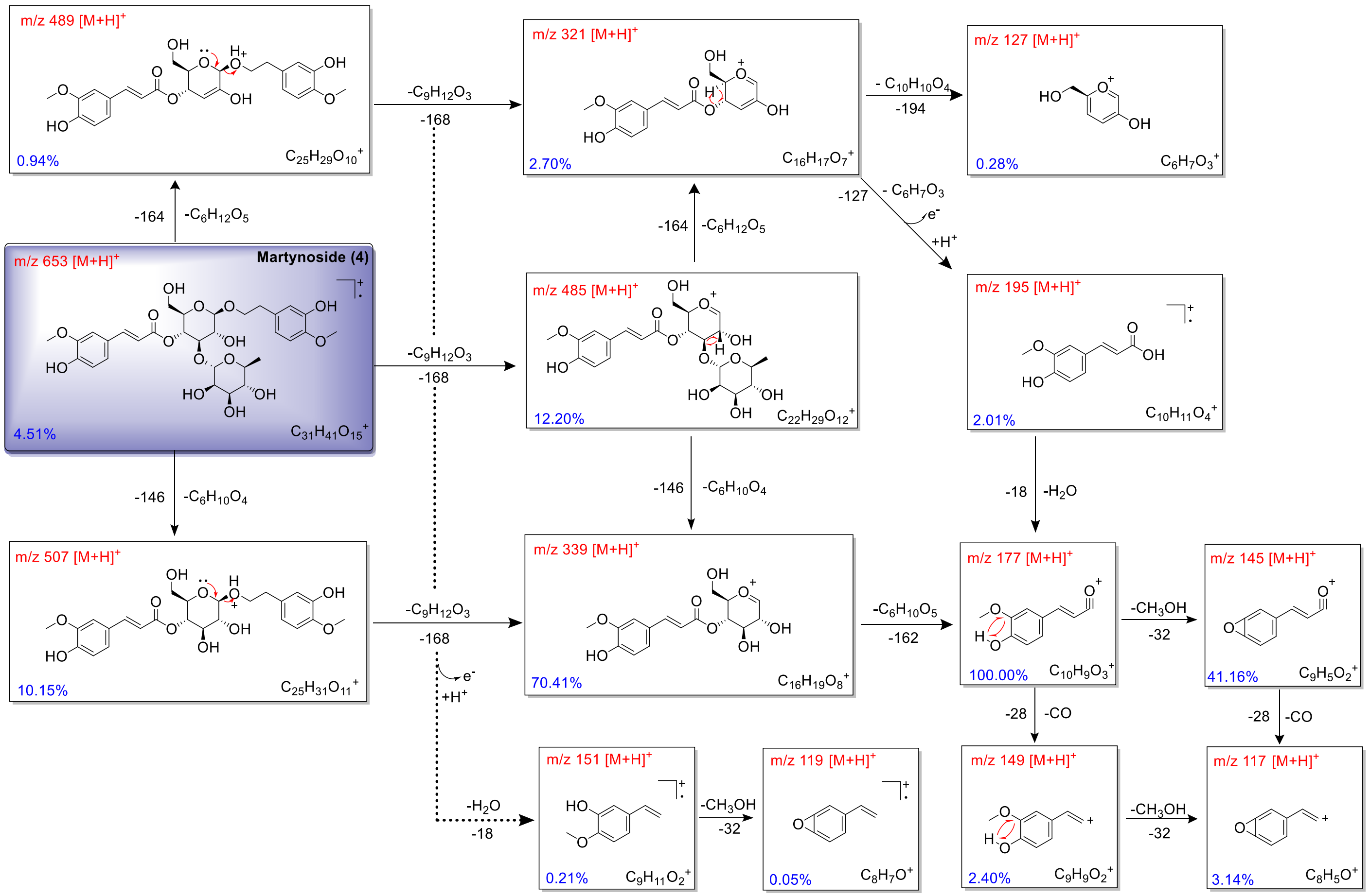

Figure S25 Possible mass fragmentation pathways of **6**.

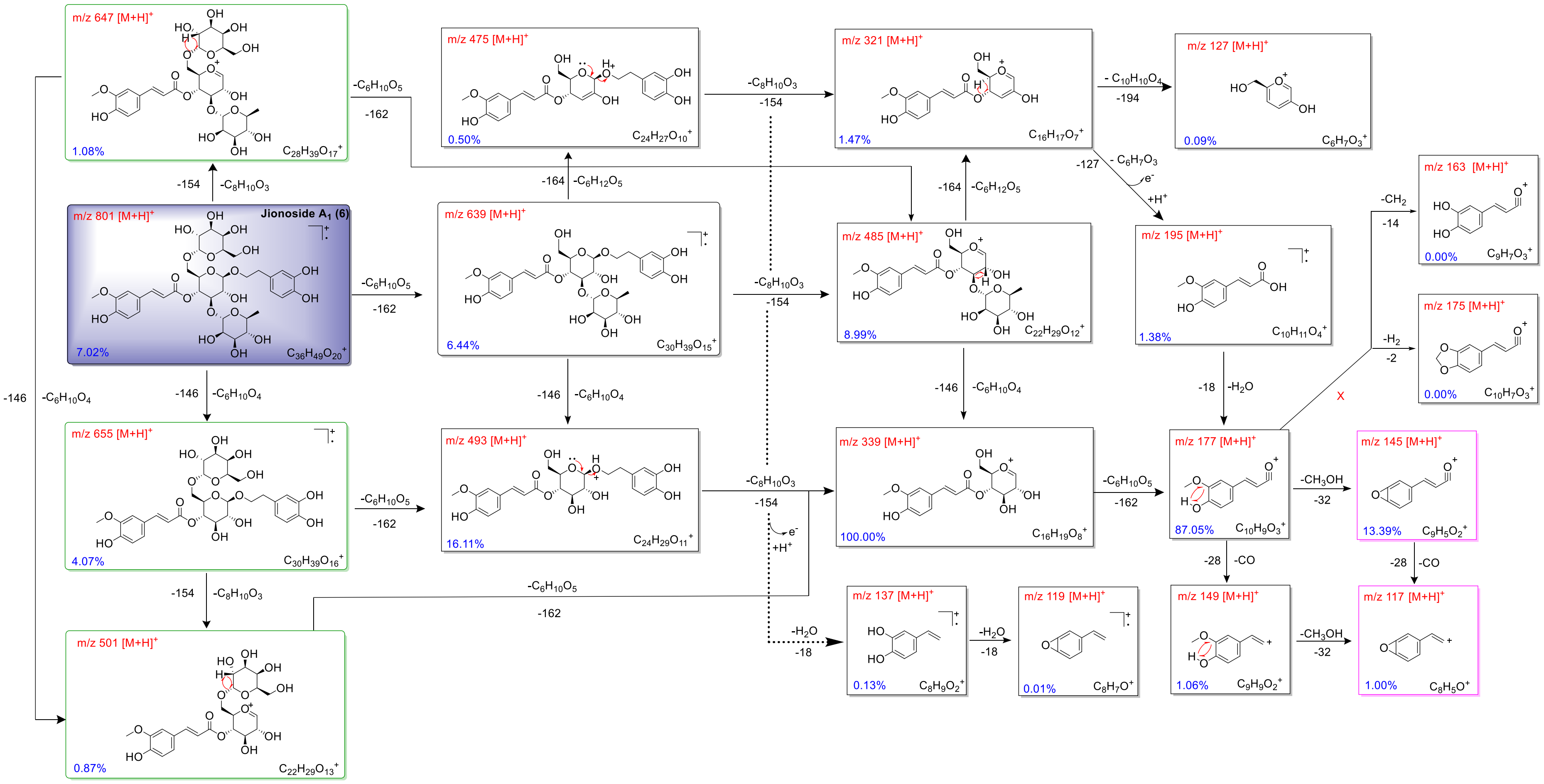

Figure S26 Possible mass fragmentation pathways of **7**.

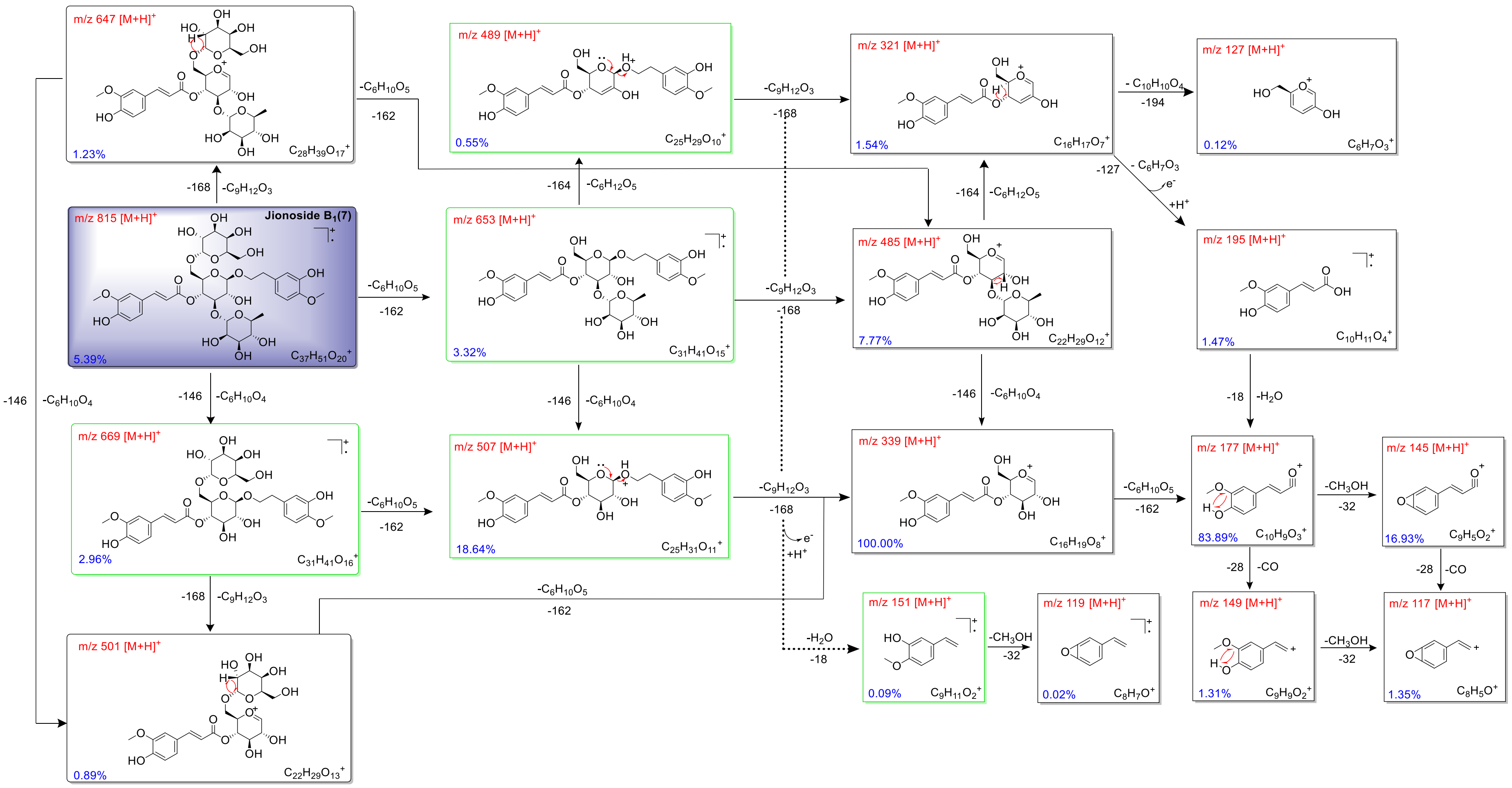

Table S1 Eremophilane-type sesquiterpenes isolated from *Rehmannia glutinosa*

No.	Name	Fomular	Structure	Source	Type	Pharmacological evaluation	Ref.
1	Remophilanetriol	$C_{15}H_{24}O_4$	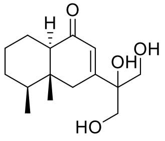	Dry radix rehmanniae	Eremophilane alcohol	——	15
2	Remophilanetriol A	$C_{15}H_{22}O_4$	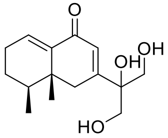	Fresh rehmannia root		The cytoprotective effect against LPS-induced injury in NRK-52E cells	18
3	Remophilanetriol B	$C_{15}H_{24}O_5$	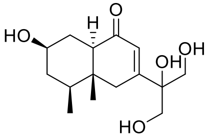	Fresh rehmannia root		The cytoprotective effect against LPS-induced injury in NRK-52E cells	40
4	Remophilanetriol H	$C_{15}H_{22}O_3$	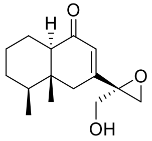	Fresh rehmannia root		Anti-pulmonary fibrosis effect	40
5	Remophilanetriol E	$C_{15}H_{20}O_3$	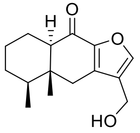	Fresh rehmannia root	Furanoeremophilane	Anti-pulmonary fibrosis effect	40
6	Remophilanetriol G	$C_{15}H_{18}O_3$	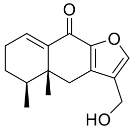	Fresh rehmannia root		Anti-pulmonary fibrosis effect	40

7	Remophilanetriol F	$C_{18}H_{24}O_5$	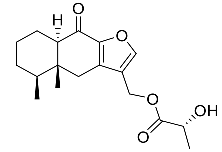	Fresh rehmannia root		Anti-pulmonary fibrosis effect	40
8	Remophilanetriol I	$C_{15}H_{20}O_5$	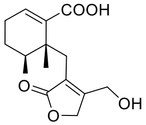	Fresh rehmannia root	Seco-eremophilane	Anti-pulmonary fibrosis effect	40
9	Serratifolide F	$C_{21}H_{30}O_{10}$	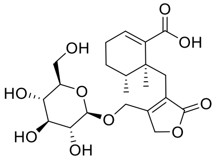	Fresh rehmannia root		The cytoprotective effect against LPS-induced injury in NRK-52E cells	44
10	Diincarvilone C	$C_{30}H_{40}O_4$	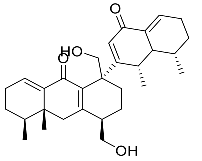	Fresh rehmannia root	Eremophilane dimer	The cytoprotective effect against LPS-induced injury in NRK-52E cells	18
11	Diincarvilone D	$C_{29}H_{36}O_4$	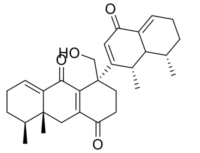	Fresh rehmannia root		The cytoprotective effect against LPS-induced injury in NRK-52E cells	18
12	Diincarvilone A	$C_{30}H_{40}O_5$	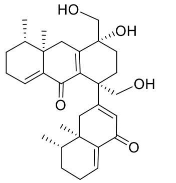	Dry radix rehmanniae		——	45

Table S2 Elemental constituents of major ions from UPLC-Q-TOF-MS/MS spectra for acteoside (**3**).

Fragment ion $[M+H]^+$	Formula	Observed	Calculated	Error (PPM)	Relative abundance
$C_{29}H_{36}O_{15}Na^+$	$C_{29}H_{36}O_{15}$	647.1910	647.1952	-6.4	12.10%
$C_{29}H_{37}O_{15}^+$	$C_{29}H_{36}O_{15}$	625.2098	625.2132	-5.4	3.48%
$C_{23}H_{27}O_{11}^+$	$C_{23}H_{26}O_{11}$	479.1587	479.1553	7.1	9.58%
$C_{21}H_{27}O_{12}^+$	$C_{21}H_{26}O_{12}$	471.1487	471.1503	-3.4	7.61%
$C_{23}H_{25}O_{10}^+$	$C_{23}H_{24}O_{10}$	461.1479	461.1448	6.7	0.87%
$C_{15}H_{17}O_8^+$	$C_{15}H_{16}O_8$	325.0889	325.0923	-10.5	65.77%
$C_{15}H_{15}O_7^+$	$C_{15}H_{14}O_7$	307.0808	307.0818	-3.3	1.76%
$C_9H_9O_4^+$	$C_9H_8O_4$	181.0512	181.0501	6.1	1.20%
$C_9H_7O_3^+$	$C_9H_6O_3$	163.0381	163.0395	-8.6	100.00%
$C_9H_5O_2^+$	$C_9H_4O_2$	145.0303	145.0290	9.0	4.62%
$C_8H_9O_2^+$	$C_8H_8O_2$	137.0594	137.0603	-6.6	0.05%
$C_8H_7O_2^+$	$C_8H_6O_2$	135.0436	135.0446	-7.4	2.40%
$C_6H_7O_3^+$	$C_6H_6O_3$	127.0384	127.0395	-8.7	0.17%
$C_8H_7O^+$	C_8H_6O	119.0473	119.0497	-20.2	0.02%
$C_8H_5O^+$	C_8H_4O	117.0320	117.0340	-17.1	1.41%
$C_{20}H_{31}O_{12}^+$	$C_{20}H_{30}O_{12}$	——	463.1816	——	0.00%
$C_{14}H_{21}O_8^+$	$C_{14}H_{20}O_8$	——	317.1236	——	0.00%
$C_{12}H_{21}O_9^+$	$C_{12}H_{20}O_9$	——	309.1186	——	0.00%
$C_{26}H_{41}O_{17}^+$	$C_{26}H_{40}O_{17}$	——	625.2344	——	0.00%

Table S3 Elemental constituents of major ions from UPLC-Q-TOF-MS/MS spectra for compound (**4**)

Fragment ion $[M+H]^+$	Formula	Observed	Calculated	Error (PPM)	Relative abundance
$C_{31}H_{40}O_{15}Na^+$	$C_{31}H_{40}O_{15}$	675.2302	675.2265	5.5	9.85%
$C_{31}H_{41}O_{15}^+$	$C_{31}H_{40}O_{15}$	653.2424	653.2445	-3.2	4.51%
$C_{25}H_{31}O_{11}^+$	$C_{25}H_{30}O_{11}$	507.1856	507.1866	-2	10.15%
$C_{22}H_{29}O_{12}^+$	$C_{22}H_{28}O_{12}$	485.1642	485.1659	-3.5	12.20%
$C_{25}H_{29}O_{10}^+$	$C_{25}H_{28}O_{10}$	489.1806	489.1761	9.2	0.94%
$C_{16}H_{19}O_8^+$	$C_{16}H_{18}O_8$	339.1089	339.1080	2.7	70.41%
$C_{16}H_{17}O_7^+$	$C_{16}H_{16}O_7$	321.0965	321.0974	-2.8	2.70%
$C_{10}H_{11}O_4^+$	$C_{10}H_{10}O_4$	195.0653	195.0657	-2.1	2.01%
$C_{10}H_9O_3^+$	$C_{10}H_8O_3$	177.0573	177.0552	11.9	100.00%
$C_9H_5O_2^+$	$C_9H_4O_2$	145.0272	145.0290	-12.4	41.16%
$C_9H_9O_2^+$	$C_9H_8O_2$	149.0604	149.0603	0.7	2.40%
$C_6H_7O_3^+$	$C_6H_6O_3$	127.0363	127.0395	-25.2	0.28%
$C_8H_5O^+$	C_8H_4O	117.0341	117.0340	0.9	3.14%
$C_9H_{11}O_2^+$	$C_9H_{10}O_2$	151.0739	151.0759	-13.2	0.21%
$C_8H_7O^+$	C_8H_6O	119.0494	119.0497	-2.5	0.05%
$C_9H_7O_3^+$	$C_9H_6O_3$	——	163.0395	——	0.00%
$C_{10}H_7O_3^+$	$C_{10}H_6O_3$	——	175.0395	——	0.00%

Table S4 Elemental constituents of major ions from UPLC-Q-TOF-MS/MS spectra for compound (**5**)

Fragment ion $[M+H]^+$	Formula	Observed	Calculated	Error (PPM)	Relative abundance
$C_{35}H_{46}O_{20}Na^+$	$C_{35}H_{46}O_{20}$	809.2474	809.2480	-0.7	12.56%
$C_{35}H_{47}O_{20}^+$	$C_{35}H_{46}O_{20}$	787.2701	787.2661	5.1	7.12%
$C_{29}H_{37}O_{16}^+$	$C_{29}H_{36}O_{16}$	641.2054	641.2082	-4.4	4.32%
$C_{27}H_{37}O_{17}^+$	$C_{27}H_{36}O_{17}$	633.2001	633.2031	-4.7	0.90%
$C_{29}H_{37}O_{15}^+$	$C_{29}H_{36}O_{15}$	625.2098	625.2132	-5.4	5.24%
$C_{21}H_{27}O_{13}^+$	$C_{21}H_{26}O_{13}$	487.1429	487.1452	-4.7	0.64%
$C_{23}H_{27}O_{11}^+$	$C_{23}H_{26}O_{11}$	479.1587	479.1553	7.1	13.69%
$C_{21}H_{27}O_{12}^+$	$C_{21}H_{26}O_{12}$	471.1487	471.1503	-3.4	7.79%
$C_{23}H_{25}O_{10}^+$	$C_{23}H_{24}O_{10}$	461.1479	461.1448	6.7	0.75%
$C_{15}H_{17}O_8^+$	$C_{15}H_{16}O_8$	325.0889	325.0923	-10.5	79.60%
$C_{15}H_{15}O_7^+$	$C_{15}H_{14}O_7$	307.0808	307.0818	-3.3	1.72%
$C_9H_9O_4^+$	$C_9H_8O_4$	181.0512	181.0501	6.1	1.22%
$C_9H_7O_3^+$	$C_9H_7O_3$	163.0381	163.0395	-8.6	100.00%
$C_9H_5O_2^+$	$C_9H_4O_2$	145.0303	145.0290	9.0	4.33%
$C_8H_9O_2^+$	$C_8H_8O_2$	137.0594	137.0603	-6.6	0.17%
$C_8H_7O_2^+$	$C_8H_6O_2$	135.0436	135.0446	-7.4	2.43%
$C_6H_7O_3^+$	$C_6H_6O_3$	127.0384	127.0395	-8.7	0.13%
$C_8H_5O^+$	C_8H_4O	117.0320	117.0340	-17.1	1.28%
$C_8H_7O^+$	C_8H_6O	——	119.0473	——	0.00%

Table S5 Elemental constituents of major ions from UPLC-Q-TOF-MS/MS spectra for compound (**6**)

Fragment ion $[M+H]^+$	Formula	Observed	Calculated	Error (PPM)	Relative abundance
$C_{36}H_{48}O_{20}Na^+$	$C_{36}H_{48}O_{20}$	823.2655	823.2637	2.2	11.46%
$C_{36}H_{49}O_{20}^+$	$C_{36}H_{48}O_{20}$	801.2867	801.2817	6.2	7.02%
$C_{30}H_{39}O_{16}^+$	$C_{30}H_{38}O_{16}$	655.2209	655.2238	-4.4	4.07%
$C_{28}H_{39}O_{17}^+$	$C_{28}H_{38}O_{17}$	647.2178	647.2187	-1.4	1.08%
$C_{30}H_{39}O_{15}^+$	$C_{30}H_{38}O_{15}$	639.2283	639.2289	-0.9	6.44%
$C_{22}H_{29}O_{13}^+$	$C_{22}H_{28}O_{13}$	501.1639	501.1608	6.2	0.87%
$C_{24}H_{29}O_{11}^+$	$C_{24}H_{28}O_{11}$	493.1745	493.171	7.1	16.11%
$C_{22}H_{29}O_{12}^+$	$C_{22}H_{28}O_{12}$	485.1639	485.1659	-4.1	8.99%
$C_{24}H_{27}O_{10}^+$	$C_{24}H_{26}O_{10}$	475.1606	475.1604	0.4	0.50%
$C_{16}H_{19}O_8^+$	$C_{16}H_{18}O_8$	339.1099	339.1080	5.6	100.00%
$C_{16}H_{17}O_7^+$	$C_{16}H_{16}O_7$	321.0967	321.0974	-2.2	1.47%
$C_{10}H_{11}O_4^+$	$C_{10}H_{10}O_4$	195.0660	195.0657	1.5	1.38%
$C_{10}H_9O_3^+$	$C_{10}H_8O_3$	177.0526	177.0552	-14.7	87.05%
$C_9H_9O_2^+$	$C_9H_8O_2$	149.0593	149.0603	-6.7	1.06%
$C_9H_5O_2^+$	$C_9H_4O_2$	145.0260	145.0290	-20.7	13.39%
$C_8H_9O_2^+$	$C_8H_8O_2$	137.0594	137.0603	-6.6	0.13%
$C_6H_7O_3^+$	$C_6H_6O_3$	127.0384	127.0395	-8.7	0.09%
$C_8H_7O^+$	C_8H_6O	119.0511	119.0497	11.8	0.01%
$C_8H_5O^+$	C_8H_4O	117.0320	117.0340	-17.1	1.00%
$C_9H_7O_3^+$	$C_9H_6O_3$	——	163.0395	——	0.00%
$C_{10}H_7O_3^+$	$C_{10}H_6O_3$	——	175.0395	——	0.00%

Table S6 Elemental constituents of major ions from UPLC-Q-TOF-MS/MS spectra for compound (**7**)

Fragment ion $[M+H]^+$	Formula	Observed	Calculated	Error (PPM)	Relative abundance
$C_{37}H_{50}O_{20}Na^+$	$C_{37}H_{50}O_{20}$	837.2819	837.2793	3.1	5.20%
$C_{37}H_{51}O_{20}^+$	$C_{37}H_{50}O_{20}$	815.2964	815.2974	-1.2	5.39%
$C_{31}H_{41}O_{16}^+$	$C_{31}H_{40}O_{16}$	669.2422	669.2395	4.0	2.96%
$C_{31}H_{41}O_{15}^+$	$C_{31}H_{40}O_{15}$	653.2493	653.2445	7.3	3.32%
$C_{28}H_{39}O_{17}^+$	$C_{28}H_{38}O_{17}$	647.2178	647.2187	-1.4	1.23%
$C_{25}H_{31}O_{11}^+$	$C_{25}H_{30}O_{11}$	507.1862	507.1866	-0.8	18.64%
$C_{22}H_{29}O_{13}^+$	$C_{22}H_{28}O_{13}$	501.1639	501.1608	6.2	0.89%
$C_{25}H_{29}O_{10}^+$	$C_{25}H_{28}O_{10}$	489.1727	489.1761	-7.0	0.55%
$C_{22}H_{29}O_{12}^+$	$C_{22}H_{28}O_{12}$	485.1639	485.1659	-4.1	7.77%
$C_{16}H_{19}O_8^+$	$C_{16}H_{18}O_8$	339.1099	339.1080	5.6	100.00%
$C_{16}H_{17}O_7^+$	$C_{16}H_{16}O_7$	321.0967	321.0974	-2.2	1.54%
$C_{10}H_{11}O_4^+$	$C_{10}H_{10}O_4$	195.0660	195.0657	1.5	1.47%
$C_{10}H_9O_3^+$	$C_{10}H_8O_3$	177.0573	177.0552	11.9	83.89%
$C_9H_{11}O_2^+$	$C_9H_{10}O_2$	151.0773	151.0759	9.3	0.09%
$C_9H_9O_2^+$	$C_9H_8O_2$	149.0593	149.0603	-6.7	1.31%
$C_9H_5O_2^+$	$C_9H_4O_2$	145.0303	145.0290	9.0	16.93%
$C_6H_7O_3^+$	$C_6H_6O_3$	127.0384	127.0395	-8.7	0.12%
$C_8H_7O^+$	C_8H_6O	119.0511	119.0497	11.8	0.02%
$C_8H_5O^+$	C_8H_4O	117.0320	117.0340	-17.1	1.35%
$C_9H_7O_3^+$	$C_9H_6O_3$	——	163.0395	——	0.00%
$C_{10}H_7O_3^+$	$C_{10}H_6O_3$	——	175.0395	——	0.00%

Table S7 IC_{50} values for cytotoxic effect of **1**–**7** on endothelial cells .

Compound	IC_{50} [log(μM)]
**1**	67.47
**2**	4.407
**3**	12.64
**4**	67.30
**5**	73.32
**6**	83.89
**7**	57.68

Table S8 Experimental concentrations of compounds (**1**–**7**) (μM).

Compound	Low dose	Medium dose	High dose
**1**	1.5625	3.125	6.25
**2**	0.39	0.78	1.5625
**3**	0.78	1.5625	3.125
**4**	1.5625	3.125	6.25
**5**	1.5625	3.125	6.25
**6**	1.5625	3.125	6.25
**7**	1.5625	3.125	6.25

Table S9 The extent to which compounds (**1**–**7**) alleviate LPS-induced impairment of HUVEC migration (alleviation percentage (%), vs. control group).

Compound	0.39 μM	0.78 μM	1.5625 μM	3.125 μM	6.25 μM
**1**	——	——	24.24 ± 8.02	42.42 ± 8.02	75.76 ± 3.03
**2**	36.00 ± 8.00	44.00 ± 18.33	80.00 ± 6.93	——	——
**3**	——	40.91 ± 7.87	50.00 ± 4.55	72.73 ± 9.09	——

**4**	——	——	32.14 ± 9.45	71.43 ± 6.19	71.43 ± 10.71
**5**	——	——	38.89 ± 5.56	44.44 ± 9.62	66.67 ± 5.56
**6**	——	——	20.00 ± 4.00	52.00 ± 8.00	80.00 ± 10.58
**7**	——	——	20.59 ± 7.78	26.47 ± 2.94	64.71 ± 20.59

Table S10 Effects of compounds (**1**–**7**) on ROS levels in LPS-induced HUVECs (ROS inhibition (%), vs. control group).

Compound	0.39 μM	0.78 μM	1.5625 μM	3.125 μM	6.25 μM
**1**	——	——	22.27 ± 3.15	37.84 ± 4.25	73.53 ± 14.07
**2**	28.52 ± 1.75	49.19 ± 25.42	74.78 ± 4.56	——	——
**3**	——	21.20 ± 6.22	42.54 ± 8.39	76.43 ± 12.32	——
**4**	——	——	33.36 ± 16.69	42.84 ± 11.52	83.63 ± 9.11
**5**	——	——	-19.89 ± 8.45	80.45 ± 10.01	87.81 ± 6.10
**6**	——	——	71.69 ± 8.75	68.55 ± 0.40	84.74 ± 15.26
**7**	——	——	28.13 ± 7.45	50.25 ± 3.05	65.84 ± 18.36

Table S11 The extent to which compounds (**1**–**7**) restore LPS-induced reduction in mitochondrial fluorescence intensity in HUVECs (via MitoTracker staining) (restore percentage (%), vs. control group).

Compound	0.39 μM	0.78 μM	1.5625 μM	3.125 μM	6.25 μM
**1**	——	——	18.15 ± 10.77	23.76 ± 13.79	71.87 ± 8.64
**2**	-6.41 ± 6.74	37.55 ± 7.46	47.48 ± 8.24	——	——
**3**	——	23.37 ± 6.25	62.17 ± 5.01	65.97 ± 7.04	——
**4**	——	——	-12.56 ± 9.65	9.40 ± 2.20	57.00 ± 21.65
**5**	——	——	-1.76 ± 14.87	26.97 ± 4.96	70.90 ± 6.63
**6**	——	——	23.27 ± 17.94	48.25 ± 2.72	83.20 ± 12.72
**7**	——	——	-3.81 ± 14.95	18.48 ± 8.60	63.44 ± 7.02

Table S12 The extent to which compounds (**1**–**7**) restore the LPS-induced reduction in mitochondrial red/green fluorescence intensity ratios in HUVECs (via JC-1 staining) (restore percentage (%), vs. control group).

Compound	0.39 μM	0.78 μM	1.5625 μM	3.125 μM	6.25 μM
**1**	——	——	20.56 ± 3.21	23.12 ± 4.73	69.78 ± 8.00
**2**	21.65 ± 4.18	77.36 ± 4.29	75.36 ± 10.82	——	——
**3**	——	12.74 ± 5.04	33.64 ± 9.77	35.03 ± 4.42	——
**4**	——	——	0.70 ± 3.35	8.17 ± 2.18	30.88 ± 3.85
**5**	——	——	40.63 ± 13.42	48.67 ± 15.03	113.96 ± 30.76
**6**	——	——	-3.29 ± 1.01	7.53 ± 1.92	69.95 ± 4.18
**7**	——	——	8.98 ± 2.55	46.10 ± 11.59	53.32 ± 6.85

Table S13 The extent to which compounds (**1**–**7**) restore the LPS-induced reduction of TOM20 protein expression in HUVECs (upregulation (%), vs. control group).

Compound	0.39 μM	0.78 μM	1.5625 μM	3.125 μM	6.25 μM
**1**	——	——	11.75 ± 14.28	57.58 ± 10.23	68.70 ± 15.57
**2**	0.57 ± 13.92	42.46 ± 14.04	62.25 ± 9.69	——	——
**3**	——	46.39 ± 22.63	69.18 ± 7.31	72.93 ± 6.51	——
**4**	——	——	16.46 ± 22.69	50.28 ± 7.84	99.78 ± 3.00

**5**	——	——	65.97 ± 21.33	89.79 ± 18.07	107.95 ± 11.11
**6**	——	——	25.58 ± 3.32	51.65 ± 16.59	65.35 ± 9.42
**7**	——	——	44.44 ± 16.82	88.62 ± 10.06	102.23 ± 6.68

Table S14 The extent to which compounds (**1**–**7**) reverse the LPS-induced upregulation of DRP1 protein expression in HUVECs (restore percentage (%), vs. control group).

Compound	0.39 μM	0.78 μM	1.5625 μM	3.125 μM	6.25 μM
**1**	——	——	4.66 ± 11.31	25.11 ± 17.47	64.82 ± 5.73
**2**	32.62 ± 4.64	50.94 ± 3.11	81.81 ± 5.68	——	——
**3**	——	43.76 ± 6.17	61.25 ± 5.60	118.04 ± 19.75	——
**4**	——	——	57.29 ± 7.99	74.82 ± 11.42	130.61 ± 10.86
**5**	——	——	29.44 ± 8.21	69.90 ± 16.52	128.22 ± 9.32
**6**	——	——	29.80 ± 9.02	37.38 ± 3.93	65.63 ± 15.48
**7**	——	——	11.09 ± 0.64	42.93 ± 13.34	82.46 ± 11.96
